# Fast Delivery of Multifunctional NIR‐II Theranostic Nanoaggregates Enabled by the Photoinduced Thermoacoustic Process

**DOI:** 10.1002/advs.202301104

**Published:** 2023-04-23

**Authors:** Huilin Xie, Chen Zhang, Tingting Li, Lianrui Hu, Jianquan Zhang, Heng Guo, Zhao Liu, Dinglu Peng, Zeshun Li, Weijun Wu, Ji Gao, Zhenyu Bi, Jinghan Wang, Pengfei Zhang, Ryan T. K. Kwok, Jacky W. Y. Lam, Zhihong Guo, Lei Xi, Kai Li, Ben Zhong Tang

**Affiliations:** ^1^ Department of Chemistry Hong Kong Branch of Chinese National Engineering Research Center for Tissue Restoration and Reconstruction The Hong Kong University of Science and Technology (HKUST) Clear Water Bay Kowloon Hong Kong 999077 China; ^2^ Shenzhen Key Laboratory of Smart Healthcare Engineering Guangdong Provincial Key Laboratory of Advanced Biomaterials Department of Biomedical Engineering Southern University of Science and Technology (SUSTech) Shenzhen Guangdong 518055 China; ^3^ Guangdong Key Laboratory of Nanomedicine Shenzhen Engineering Laboratory of Nanomedicine and Nanoformulations CAS‐HK Joint Lab for Biomaterials Research Laboratory for Biomedical Optics and Molecular Imaging Shenzhen Key Laboratory for Molecular Imaging CAS Key Lab for Health Informatics Shenzhen Institutes of Advanced Technology Chinese Academy of Sciences Shenzhen Guangdong 518055 China; ^4^ School of Science and Engineering Shenzhen Institute of Aggregate Science and Technology The Chinese University of Hong Kong Shenzhen Guangdong 518172 China; ^5^ Center for Aggregation‐Induced Emission South China University of Technology (SCUT) Guangzhou Guangdong 510640 China

**Keywords:** aggregation‐induced emission, nanoaggregate fast delivery, photoinduced thermoacoustic process, phototheranostic agent

## Abstract

Multifunctional nanoaggregates are widely used in cancer phototheranostics. However, it is challenging to construct their multifunctionality with a single component, and deliver them rapidly and efficiently without complex modifications. Herein, a NIR‐absorbing small molecule named TBT‐2(TP‐DPA) is designed and certify its theranostic potentials. Then, their nanoaggregates, which are simply encapsulated by DSPE‐PEG, demonstrate a photothermal efficiency of 51% while keeping a high photoluminescence quantum yield in the NIR region. Moreover, the nanoaggregates can be excited and delivered by an 808 nm pulse laser to solid tumors within only 40 min. The delivery efficiency and theranostic efficacy are better than that of the traditional enhanced permeability and retention (EPR) effect (generally longer than 24 hours). This platform is first termed as the photoinduced thermoacoustic (PTA) process, and confirm its application requires both NIR‐responsive materials and pulse laser irradiation. This study not only inspires the design of multifunctional nanoaggregates, but also offers a feasible approach to their fast delivery. The platform reported here provides a promising prospect to boost the development of multifunctional theranostic drugs and maximize the efficacy of used medicines for their clinical applications.

## Graphical abstract

A NIR‐II‐emissive AIEgen is designed by introducing appropriate *π*‐bridges into the D‐A‐D molecule, and its multifunctional nanoaggregates are facilely constructed. The delivery of the nanoaggregates to the tumor sites is accelerated by the photoinduced thermoacoustic (PTA) process within 40 min. Hence, the phototheranostic efficacy is significantly improved compared with the traditional enhanced permeability and retention (EPR) effect for 24 h.

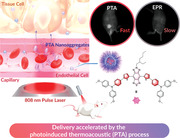

## 1 Introduction

Nanoaggregates, including nanoparticles and the cluster of molecules, have aroused much research interest due to their distinct properties compared with materials at the molecular level.^[^ ^1^ ^]^ For example, the nanocrystals of drugs can conquer the issues of insolubility and bioavailability, which have better effects than single molecules in clinics.^[^ ^2^ ^]^ The nanoaggregates of molecules with aggregation‐induced emission (AIE) characteristics can emit much brighter luminescence compared to their dispersed solutions, which has advantages in imaging‐guided diagnostics and therapies, and fabrication of optoelectronic devices during the past 20 years.^[^ ^1^, ^3^ ^]^ Aggregates are especially important in multifunctional materials because they may be the smallest entities that are genuinely working.^[^ ^3^, ^4^ ^]^ Aggregates can either be homo‐ or heterogeneous. Homo‐aggregates are constituted by a single component that can exhibit distinct properties from single molecules by different packing modes, where different stacking of the nanoaggregates can perform various duties.^[^ ^5^ ^]^ Hetero‐aggregates can offer more property diversities because of the richer interactions between the multiple components, but the factual outcomes are hard to anticipate due to the complexity of these systems. Hence, regulating the multifunctionality of nanoaggregates is of vital significance to practical applications.

Theranostics commonly require multifunctional agents, which is difficult to achieve in single molecules. Recently, emerging phototheranostics of cancer nanomedicine are hot topics,^[^ ^6^ ^]^ because utilizing the energy of photoirradiation, theranostic agents can ablate cancer cells under the guidance of single or multimodal imaging, facilitating cancer diagnosis and treatment simultaneously.^[^ ^7^ ^]^ Traditionally, several components with individual functions, such as drugs and imaging probes, are packaged in one nanoparticle as multifunctional nanomedicine.^[^ ^6^, ^8^ ^]^ However, the complexity and cost of this “all‐in‐one” manner have limited their development for clinical use. Homo‐aggregates derived from a single component are thus highly demanded, in which their properties and functionalities are controllable and predictable. AIE luminogen (AIEgens) are ideal design templates for the construction of phototheranostic agents in a “one‐for‐all” manner because on the one hand, they can overcome the aggregation‐caused quenching (ACQ) problem^[^ ^3b^ ^]^ and the bright fluorescence can still be maintained in the aggregated state to achieve stable, long‐lasting tracking of bioprocesses. Especially, via rational design, AIEgens can be tuned to absorb and emit near‐infrared (NIR) light (780–1700 nm) to enjoy the merits of less autofluorescence and deeper penetration depth than the contrast agents excited by visible light,^[^ ^7^, ^9^ ^]^ which is more realistic for biomedical applications. On the other hand, the energy decay pathways of aggregates are more complicated, which offers more possibilities for desirable energy distribution for constructing one‐for‐all theranostic agents. Moreover, the active nonradiative decay in the NIR‐II region is usually a trade‐off for heat dissipation, which can be utilized for photothermal therapy and photoacoustic response.^[^ ^10^ ^]^ The formation of nanoaggregates can then trigger a synergistic effect to generate more heat for cancer ablation and other functions. Therefore, NIR‐II emissive nanoaggregates exhibiting multifunctionalities and multiplex applications such as fluorescence imaging and theranostics capability are of bright prospects in the biological and clinic fields.

Another critical problem restricting the effective utilization of theranostic agents is their delivery efficiency. Although the enhanced permeability and retention (EPR) effect has been widely acknowledged for decades, only ≈ 0.7% (median) of the administered dose can be delivered to solid tumors.^[^ ^11^ ^]^ In addition, it requires 24 h or longer time for EPR or other specific targeting modifications to accumulate sufficiently.^[^ ^12^ ^]^ To address this issue, photoacoustic radiation force has been discovered^[^ ^13^ ^]^ and recently applied to in vivo drug delivery, which is more efficient compared with the individual EPR process.^[^ ^14^ ^]^ Utilizing the photoinduced thermoacoustic (PTA) process, theranostic agents can be excited and delivered to solid tumors within one hour.^[^ ^14a^ ^]^ The delivery, accelerated by near‐infrared pulse laser irradiation, is a simple physical way without any other chemical or biological modification,^[^ ^14a,b^ ^]^ which can reduce the waste of theranostic nanoaggregates caused by the inefficient accumulation in clinical trials.

**Figure 1:**
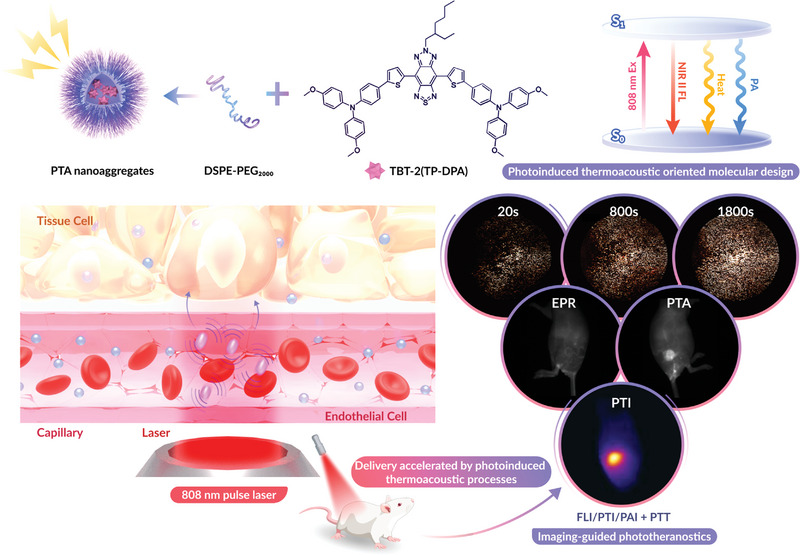
A schematic diagram of the NIR‐II theranostic nanoaggregates construction and their fast delivery enabled by photoinduced thermoacoustic (PTA) processes for multifunctional imaging and enhanced photothermal therapy. Top: Photoinduced thermoacoustic oriented molecular design for construction of NIR‐II AIEgen and illustration of the reconciled photophysical processes of the TBT‐2(TP‐DPA) PTA nanoaggregates after photoexcitation. Bottom: Applications of PTA nanoaggregates in the PTA process: accelerated nanoaggregates delivery and multifunctional FLI/PTI/PAI imaging‐guided phototheranostics.

Herein, we develop a series of D‐(*π*)‐A‐(*π*)‐D organic small molecules with well‐designed *π*‐bridges, named TBT‐2(1P‐DPA), TBT‐2(2P‐DPA), and TBT‐2(TP‐DPA), as the candidates for one‐for‐all phototheranostic agents. It turns out that TBT‐2(TP‐DPA) with the thiophene‐containing bridge exhibits the most balanced NIR imaging and therapeutic potential. The calculation results suggest that TBT‐2(TP‐DPA) shows the strongest D‐A interaction and the favorable intramolecular charge transfer effect, which benefits the longer emission wavelength extended to ≈ 1500 nm. After simple encapsulation with 1, 2‐distearoyl‐sn‐glycero‐3‐phosphoethanolamine‐poly(ethylene glycol) (DSPE‐PEG), the nanoaggregates of TBT‐2(TP‐DPA) can demonstrate photothermal conversion efficiency of up to 51% while keeping a high photoluminescence quantum yield in the NIR region as well. These phototheranostic nanoaggregates can achieve multimodal imaging of fluorescence imaging (FLI), photothermal imaging (PTI), and photoacoustic imaging (PAI). Meanwhile, they can be applied to enhance photothermal therapy (PTT) efficiency and eliminate solid tumors effectively in the 4T1‐tumor‐bearing mouse model via PTA process (**Figure** **1**). For the first time, the PTA process based on NIR‐absorbing one‐for‐all phototheranostic nanoaggregates of small molecules enables fast drug delivery in vivo for highly efficient cancer therapy. This work not only proposes rational design strategies for multifunctional theranostic nanoagents, but also provides a practical way for their rapid delivery to the targeted tissue by utilizing the PTA process.

## 2 Results and Discussion

**Figure 2:**
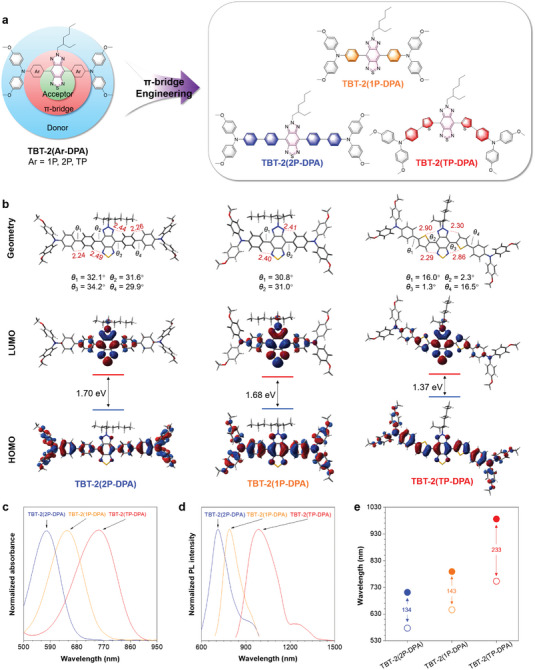
Molecular structures and photophysical details. a) Molecular design principle and chemical structures of TBT‐2(1P‐DPA), TBT‐2(2P‐DPA), and TBT‐2(TP‐DPA). b) Optimized S_0_ geometries and illustration of the frontier molecular orbitals (LUMOs and HOMOs) determined by the B3LYP/6‐31G* level of theory, unit: Å. c) Normalized absorption spectra and (d) normalized photoluminescence (PL) spectra of the compounds in THF solution (10 µM). (e) Plots of absorption maximum (hollow circle) and emission maximum (solid circle) in THF and the corresponding Stokes shift of the three compounds.

Developing phototheranostic agents with bright NIR‐II fluorescence is of vital significance, but the majority of reported NIR‐II‐emissive molecules exhibit low fluorescence quantum yield (QY).^[^ ^10b^ ^]^ Here, we applied a strong electron‐withdrawing unit named thiadiazolobenzotriazole (TBT) as the acceptor core to design a series of NIR‐emissive molecules. Starting from diphenylamine (DPA) as the electron‐donating unit, we employed three different aromatic moieties, namely phenyl, biphenyl, and phenylthienyl groups, as the *π*‐units to bridge the TBT acceptor and the DPA donor. Despite the simple structures of these *π*‐units, it is anticipated that they endow the molecules with various electronic effects and aggregation behavior when incorporated into the D‐*π*‐A‐*π*‐D molecules.^[^ ^9^, ^15^ ^]^ The three final products, TBT‐2(1P‐DPA), TBT‐2(2P‐DPA), and TBT‐2(TP‐DPA), can be synthesized via facial Stille coupling reactions with high yield and easy purification, as shown in **Figure** **2**a. The intermediates and final products were all determined by ^1^H and ^13^C nuclear magnetic resonance (NMR) and matrix‐assisted laser desorption ionization‐time of flight (MALDI‐TOF) mass spectrometer (Figures S1–S13, Supporting Information).

Density‐functional theory (DFT) calculations with B3LYP functional and 6–31G* basis was set to obtain the optimized geometries and molecular orbitals of TBT‐2(1P‐DPA), TBT‐2(2P‐DPA), and TBT‐2(TP‐DPA) (Figure 2b). As for the optimized geometries, TBT‐2(1P‐DPA) and TBT‐2(2P‐DPA) with the phenyl rings directly linked to the TBT unit exhibit twisted molecular backbone, where the dihedral angles between the TBT unit and the phenyl rings are 30–35°. Moreover, the two phenyl rings in the *π*‐bridges of TBT‐2(2P‐DPA) also form a twisting angle of ≈ 30° due to the steric hindrance. In comparison, the thiophene rings of TBT‐2(TP‐DPA) generate much smaller dihedral angles of ≈ 2° and ≈ 16° with the adjacent TBT unit and phenyl rings, respectively, resulting in a coplanar central part and twisted wings of the molecule. Consequently, the different planarity of these molecules significantly affects the conjugation and D‐A interactions. It can be observed from the highest occupied molecular orbital/lowest unoccupied molecular orbital (HOMO/LUMO) distributions that TBT‐2(1P‐DPA) shows typical D‐A characters, which can be seen as the reference. The additional phenyl rings of TBT‐2(2P‐DPA) block the conjugation between the DPA and TBT units, where no LUMO locates at the N atoms at the DPA units. On the contrary, the thiophene rings of TBT‐2(TP‐DPA) not only extend the conjugation length owing to the fewer twisting angles but also strengthen the intramolecular charge transfer (ICT) due to their more electron richness. As a result, the HOMO‐LUMO gaps of the molecules follow the sequence of TBT‐2(2P‐DPA), TBT‐2(1P‐DPA), and TBT‐2(TP‐DPA), more specifically, 1.70, 1.68, and 1.37 eV, respectively. The calculation results indicate that the *π*‐bridges can alter the electronic structures of the molecules, which in turn regulate the optical properties of the materials.

To testify the molecular simulation results, we then measured the absorption and emission spectra of TBT‐2(1P‐DPA), TBT‐2(2P‐DPA), and TBT‐2(TP‐DPA) in various solvents. It turns out that TBT‐2(1P‐DPA) and TBT‐2(2P‐DPA) possess absorption peaks at 650 and 580 nm in THF solution, respectively, and their absorption both ended up to 800 nm (Figure 2c). However, the absorption peak of TBT‐2(TP‐DPA) was ≈ 760 nm, and the whole absorption wavelength can be extended to NIR‐I region (Figure 2c), which shows potential as an agent for NIR excitation. Expectedly, the emission spectrum of TBT‐2(1P‐DPA), and TBT‐2(2P‐DPA) both ends below 1000 nm, while the emission maximum of TBT‐2(TP‐DPA) was ≈ 1000 nm when upon 808 nm excitation (Figure 2d). These results indicated that with the decrease of twisted degree, the absorption and emission spectrum of TBT‐2(2P‐DPA), TBT‐2(1P‐DPA), and TBT‐2(TP‐DPA) presented an increasing trend in wavelengths (Figure 2c,d), among which TBT‐2(TP‐DPA) displayed the largest Stokes shift (Figure 2e). The experimental characterization is consistent with our theoretical hypothesis, which made TBT‐2(TP‐DPA) an ideal candidate as a NIR‐II imaging agent.

Moreover, red‐shifted maximum absorption wavelengths can be observed when the polarity of solvents increases, indicating the twisted intramolecular charge transfer (TICT) effect of the three designed fluorophores (Figures S14–S16, Supporting Information). The emission spectrum of TBT‐2(TP‐DPA) in various solvents also demonstrated its apparent TICT effects even in the NIR‐II region (Figure S17, Supporting Information). We then tested the AIE properties of the fluorophores. With the increment of water fraction in the THF/water system, all the systems exhibited fluorescence reduction at a low water ratio first, then an enhancement at a higher water ratio, suggesting the exist of AIE and TICT effect (Figures S18–S23, Supporting Information). Interestingly, although the AIE phenomena of TBT‐2(TP‐DPA) in THF/water solution is not so obvious (Figure S22 and S23, Supporting Information), when the mixture is changing to chloroform/hexane, we can again observe the typical AIE curve of the molecule (Figure S24 and S25, Supporting Information). The different phenomena are due to the poor solubility of TBT‐2(TP‐DPA) in the THF/water system..

Under 808 nm excitation, the quantum yield of TBT‐2(TP‐DPA) in the NIR region was determined to be as high as 6.0% in toluene solution when taking IR 26 in dichloroethane (0.05%) as a reference (Figure S26, Supporting Information). The value of the calculated relative quantum yield is within the range of error when compared to the absolute QY value tested by the integrating sphere (10.4% in toluene) (Table S1, Supporting Information). The QY values of the molecule in other solvents and in the solid state all indicated its superior fluorescence emission capability in the NIR region (Table S1, Supporting Information), making it a qualified probe for in vivo imaging.

**Figure 3:**
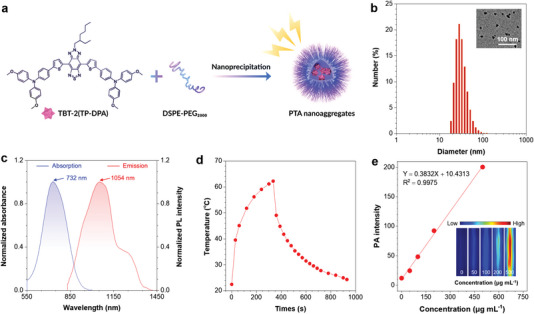
a) The schematic illustration of the preparation of PTA nanoaggregates. b) DLS distribution of the PTA nanoaggregates in water. Inset: TEM image of the nanoaggregates. c) Normalized absorption and photoluminescence (PL) spectra of the PTA nanoaggregates in aqueous medium. d) Photothermal conversion curve of the PTA nanoaggregates. e) The photoacoustic (PA) spectrum of the PTA nanoaggregates in an aqueous solution. Insets: photoacoustic images of PTA nanoaggregates at concentrations of 0, 50, 100, 200, and 500 µg mL^−1^.

According to Jablonski diagrams, the acquisition of NIR fluorophores is always a win‐win strategy for phototheranostics because we can make the best use of the nonradiative decay for multimodal theranostics, such as photothermal or photoacoustic imaging and related therapy.^[^ ^10^, ^16^ ^]^ In biological applications, polymeric nanoprecipitation is commonly used to bring hydrophobic molecules into biological media. Among them, DSPE‐PEG is widely used in drug delivery.^[^ ^17^ ^]^ Its biocompatibility, biodegradability, and amphiphilicity have made them ideal nanoparticle shells for encapsulating theranostic agents including AIEgens.^[^ ^3^, ^18^ ^]^ Such encapsulation can, in turn, trigger the restriction of intermolecular motion, endowing AIEgens with stronger fluorescence emission when aggregated. Furthermore, thanks to the soft surfactants, the inner parts of the nanoaggregates are tightly packed while the outer part molecules close to the surface of the nanoparticles are loosely packed, which is versatile and feasible for aggregates to achieve radiative and nonradiative decay simultaneously.^[^ ^19^ ^]^ Inspired by the previous design, we simply encapsulated TBT‐2(TP‐DPA) with DSPE‐PEG_2000_ (**Figure** **3**a). The nanoaggregates of TBT‐2(TP‐DPA) were prepared with an average size of ≈ 32 nm and evenly morphology shown by dynamic laser scanning (DLS) and transmission electron microscopy (TEM) (Figure 3b and Figure S27, Supporting Information). The absorption and emission spectra of the nanoaggregates were recorded (Figure 3c), showing a maximum NIR absorption of 732 nm, and a NIR‐II emission peaked at 1054 nm, which is applicable to apply the NIR light to irradiate and obtain a deeper penetration depth for the powerful bioimaging. Moreover, the stability of TBT‐2(TP‐DPA) nanoaggregates in both 1× PBS and complete cell culture medium after continuous incubation for 7 days at 37 °C were investigated via DLS. Due to protein adsorption in the complete culture medium, the initial average diameters of the nanoaggregates in the culture medium is slightly larger than that in PBS. However, there was no significant difference in the diameters of the nanoagregates at day 0, day 1, and day 7 in either PBS or culture medium (Figure S28, Supporting Information), suggesting their excellent stability for in vivo studies.

To test the multifunctionality potentials of the nanoaggregates, heat generation capability was first evaluated by calculating their photothermal conversion efficiency (PCE). As expected, the TBT‐2(TP‐DPA) nanoaggregates performed good photothermal conversion capability, with temperature increasing to above 60 °C when suspended in water at the concentration of 1 mg mL^−1^ upon 5 min 808 nm continuous laser irradiation (Figure 3d). The PCE value of the nanoaggregates above was calculated to be 51% (Figure S29, Supporting Information). When applying a pulse laser, the photoacoustic response was prompted due to the photoinduced thermoacoustic process. The photoacoustic spectrum of nanoaggregates in water was measured to be consistent with that of absorption properties, which range from 650 to 1000 nm and has strong photoacoustic signals at 808 nm (Figure S30, Supporting Information). Particularly, the photoacoustic brightness increases gradually with the increase of nanoaggregates concentration (Figure 3e, inset). The linear relationship between photoacoustic signals and the nanoaggregate concentrations can ensure the rationality of quantifying nanoaggregate accumulation via PAI (Figure 3e). These characteristics can initially satisfy the needs for photothermal imaging, photoacoustic imaging, and photothermal therapy.

**Figure 4:**
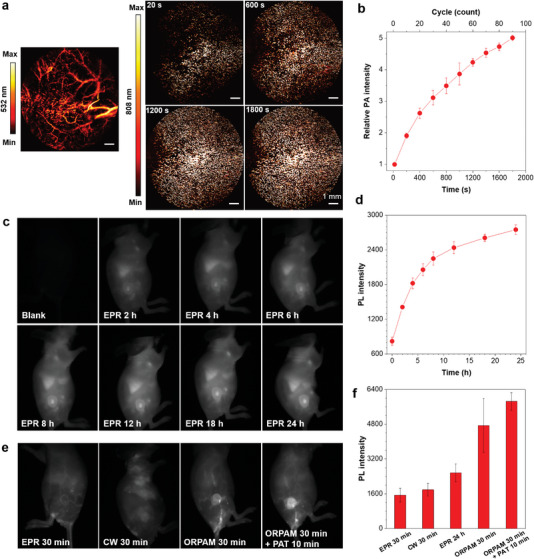
a) The dual‐wavelength (532 nm and 808 nm) ORPAM imaging of the tumor from a representative mouse intravenously injected with TBT‐2(TP‐DPA) PTA nanoaggregates (1 mg mL^−1^, 200 µL), followed by a series of sequential scanning, scale bars: 1 mm. b) Quantitative analyses of the accumulation of the nanoaggregates in the flank tumors by the photoacoustic intensity in panel a. c) The NIR‐II fluorescence imaging of tumor in EPR group post intravenous injection of PTA nanoaggregates (1 mg mL^−1^, 200 µL). d) Quantitative analyses of the time‐dependent nanoaggregates accumulation in the flank tumors in the EPR group by fluorescence intensity in panel c. e) The NIR‐II fluorescence imaging of bilateral tumors post intravenous injection of the prepared nanoaggregates (1 mg mL^−1^, 200 µL). The right tumor was treated with an 808 nm pulse laser (ORPAM and ORPAM + PAT group) or CW laser, while no treatment was performed on the left tumor. f) Quantitative analyses of the accumulation of the nanoaggregates in the flank tumors by fluorescence intensity in panels c) and e).

It is worth mentioning that in nonradiative decay, the acoustic wave from heat generation can not only assist PAI, but also be a guiding force for particle delivery in vitro and in vivo.^[^ ^13^, ^14^ ^]^ Moreover, it has been validated that all kinds of nanoparticles with the photoacoustic response, no matter small molecules like Indocyanine green (ICG), polymers, or AuNPs, can be triggered by this photoacoustic radiation force.^[^ ^14a^ ^]^ Hence, we evaluated the delivery possibilities and efficiency enabled by photoinduced thermoacoustic (PTA) processes of TBT‐2(TP‐DPA) nanoaggregates in vivo. Bilateral xenografted 4T1 tumor‐bearing mice were employed as the solid tumor models. A dual‐wavelength optical‐resolution photoacoustic microscopy (ORPAM) system was set up to assist the nanoaggregates delivery towards tumor (808 nm pulse laser), and to simultaneously visualize the structure of blood vessels (532 nm) and nanoaggregates accumulation (808 nm) in or around tumor sites. After intravenous injection of the nanoaggregates, we took both tumors on the same mouse as the PTA experimental group and the EPR control group. On the side of the PTA tumor, we established a sequential laser scanning for 30 min (20 s per cycle) using the 808 nm pulse laser to deliver nanoaggregates by PTA processes. As is seen in **Figure** **4**a, the nanoaggregates accumulated more and more in the solid tumor over time, and almost distributed spread the area when scanned for 30 min, which is highly effective. Quantitative results of the time‐dependent nanoaggregates accumulation within half an hour by photoacoustic signals can be seen in Figure 4b.

To study the precision of this PTA‐enabled delivery, we also imaged a larger field of view using NIR‐II fluorescence emitting from the nanoaggregates. On the EPR side, there was a nearly negligible fluorescent signal in the tumor after the 30 min interval. However, the fluorescence on the PTA‐treated side (ORPAM 30 min) of the same mouse can clearly distinguish the tumor and surrounding area. Further, because ORPAM is a microscope‐based scanning method, it can only scan the surface of the tumor. To evaluate the nanoaggregate accumulation in the whole tumor, we applied photoacoustic tomography (PAT) to trigger the accumulation of deeper tissue.^[^ ^20^ ^]^ The experimental results show that an even stronger fluorescent signal came out after the addition of 10 min laser scanning by PAT (ORPAM 30 min + PAT 10 min) (Figure 4e). Since the nanoaggregates are only encapsulated with DSPE‐PEG without any other targeting modification, the results can validate the feasibility of PTA‐driven delivery of the TBT‐2(TP‐DPA) nanoaggregates because PTA process can enhance vascular permeability in the tumor tissue.^[^ ^14^ ^]^ Next, to compare the effectiveness of nanoaggregates accumulation in EPR and PTA effect, we then administered the same dose of nanoaggregates via tail vein injection and observed the time‐dependent fluorescence signals in another group of mice. Under barely EPR effect, the fluorescence emission in the tumor reached its maximum at 24 h postinjection (Figure 4c,d), but it was still much weaker than that of the PTA group within 40 min (Figure 4e,f). Quantitative results demonstrated that the signal intensities in the PTA groups were nearly 3–4 times higher than that in the EPR groups in the same time slot. The fluorescence signal intensity from the ORPAM 30 min + PAT 10 min group was still as 2–3 fold as the EPR group when giving them 24 h to accumulate (Figure 4f), proving the high efficiency of this PTA‐enabled delivery manner. Notably, in this study, for the first time, we evaluate the delivery possibility of continuous laser energy towards solid tumors, by irradiating mice tumors for 30 min upon 808 nm continuous wave (CW) laser. Fluorescence signals from the CW group indicated that the continuous wave does offer a bit more energy to promote vascular permeability for accumulating particles. However, the outcome is just slightly different from the EPR group and far below the delivery efficiency of the pulse laser. It suggested that the transient enhancement of vascular permeability can be only triggered by the photoinduced thermoacoustic process occupied by the pulse laser, excluding the interference of the continuous heat generation.

**Figure 5:**
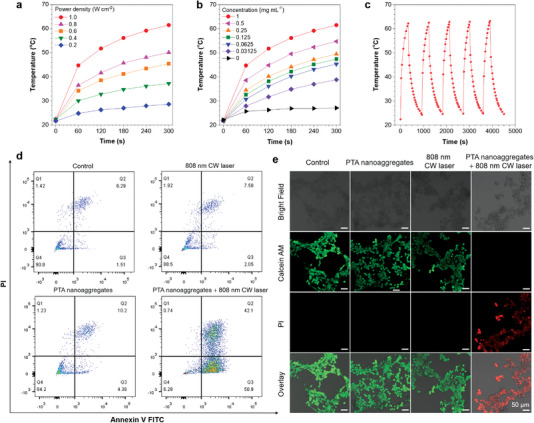
In vitro photothermal performance of the PTA nanoaggregates. a) The temperature increases of PTA nanoaggregates with the different power densities (from lower to upper: 0.2, 0.4, 0.6, 0.8 and 1 W cm^−2^) under 808 nm laser irradiation for 5 min. b) The temperature increases of the nanoaggregates with different concentrations (from lower to upper: 0, 0.03125, 0.0625, 0.125, 0.25, 0.1 and 1 mg mL^−1^) under 808 nm laser irradiation (1 W cm^−2^) for 5 min. c) Photothermal conversion curves in five NIR laser on/off cycles (808 nm). d) Flow cytometry analysis of 4T1 cells after various treatments, including cells without treatment (control), PTA nanoaggregates‐treated cells (PTA nanoaggregates), 808 nm CW laser‐treated cells (808 nm CW laser), and cells treated with PTA nanoaggregates and 808 nm CW laser (PTA nanoaggregates + 808 nm CW laser. e) Live/dead assay results of 4T1 cells after varied treatments as in d. Laser power: 1 W cm^−2^. Scale bars: 50 µm.

Based on the tuning of the TICT effect on molecular design, it is easier for NIR fluorophores to generate heat.^[^ ^10^, ^21^ ^]^ The photothermal effect of the TBT‐2(TP‐DPA) nanoaggregates depends on power density (**Figure** **5**a) and concentration (Figure 5b and Figure S31, Supporting Information), which provided the basis for optimization of the next in vitro experiments. It is noteworthy that even when experiencing continuously cyclic 5 heating/cooling processes, the nanoaggregates still exhibited excellent photostability (Figure 5c). To act as a qualified theranostic agent, the materials should possess no cytotoxicity when the laser is off. Therefore, we evaluate the dark toxicity of the nanoaggregates on 4T1 cells with CCK‐8 assay. It shows that the viabilities of 4T1 cells can keep over 80%, even at concentration of 250 µg mL^−1^ when incubated with TBT‐2(TP‐DPA) nanoaggregates for 24 h, and no higher than 50 µg mL^−1^ for 48 and 72 h, presenting good biocompatibility. (Figure S32, Supporting Information). Besides, the nanoaggregates also displayed negligible cytotoxicity towards normal cells like 293T cells, which ensured their biosafety at the cellular level (Figure S33, Supporting Information). However, when subsequently irradiated with an 808 nm continuous laser for 10 min, the cells incubated with the nanoaggregates showed apoptosis. Flow cytometry results indicated that ≈ 42.1% of the cells would experience late apoptosis and 50.9% were early apoptotic after the as‐described treatment (Figure 5d). Meanwhile, the apoptotic cell percentage of the control group, the 808 nm laser irradiation group, and the barely nanoaggregates without irradiation group were all below 15%. The significant cell‐killing effect via photothermal treatment can guarantee effective cancer cell ablation for later in vivo experiments. We also visualized the cell morphology under the PTT treatment using Calcein AM and propidium iodide (PI) as live/dead cell assay. Confocal imaging revealed that the TBT‐2(TP‐DPA) nanoaggregates + laser group emitted strong red fluorescence for dead cells, while the other groups including the control only emitted green fluorescence representing live cells (Figure 5e and Figure S34, Supporting Information), suggesting the consistency and reliability of our in vitro PTT results.

**Figure 6:**
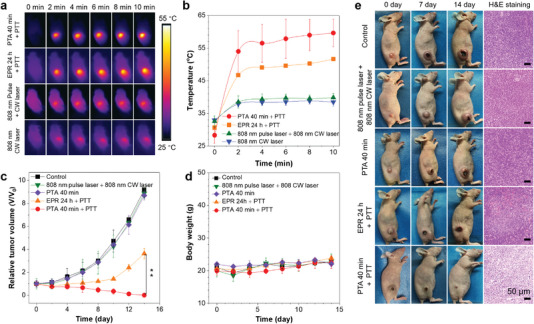
PTT efficacy of PTA nanoaggregates against subcutaneous 4T1 tumors in vivo. a) IR thermal images of 4T1 tumor‐bearing mice under an 808 nm (1 W cm^−2^) laser irradiation for 10 min after various treatments. b) The corresponding temperature change curves at the tumor site of 4T1 tumor‐bearing mice in each group. c) Tumor growth and d) body weight curves of different groups of mice. e) Representative digital images of mice in each experimental group at different time points and corresponding H&E staining of tumor sections. Data represent as mean ± s.d. (*n* = 3 mice for all groups); 0.01 < *P < 0.05, 0.001 < **P < 0.01, ***P < 0.001. Scale bars: 50 µm.

After the confirmation of nanoaggregate accumulation enabled by photoinduced thermoacoustic process and the aggregates’ photothermal effect on cancer cells, we then investigated their therapeutic effect in vivo. Several 4T1 tumor‐bearing mice were randomly divided into 5 groups. 1) TBT‐2(TP‐DPA) nanoaggregates + 808 nm pulse laser + 808 nm CW laser (PTA 40 min + PTT); 2) TBT‐2(TP‐DPA) nanoaggregates + 808 nm CW laser (EPR 24 h + PTT); 3) TBT‐2(TP‐DPA) nanoaggregates + 808 nm pulse laser (PTA 40 min); 4) 808 nm pulse laser + 808 nm CW laser; and 5) PBS (control). In this project, the aim of the 808 nm pulse laser was used in photoacoustic imaging and fast delivery (PTA 40 min), while the 808 nm CW laser was utilized for photothermal therapy (PTT, with irradiation time of 10 min). The whole therapy process can be divided into two steps. First, nanoaggregates delivery, no matter via PTA processes upon pulse laser scanning for 40 min or via EPR effect for 24 h. Then, after the nanoaggregates were accumulated enough towards the solid tumor area, the photothermal therapy will be excited by 808 nm CW laser with 10 min irradiation. To visualize the actual photothermal effect in vivo in real‐time, IR thermal camera was set up to monitor the IR imaging on all the mice in group 1), 2), 4), and 5) (**Figure** **6**a). The tumor temperature in the PTA + PTT group was elevated to 53 °C after only 2 minutes of 808 nm laser irradiation, and further rise to nearly 60 °C in the following 8 minutes (Figure 6b). In comparison, despite we leave 24 h for the EPR group to accumulate sufficient nanoaggregates toward the tumor, they only climbed to 51 °C within 10 min under the same laser treatment (Figure 6b). On the contrary, no matter whether treated with barely 808 nm CW laser (group 5, control) or plus 808 nm pulse laser (group 4), if there were no nanoaggregates injection, the laser themselves would cause slightly negligible temperature change on the tumors from the initial mice body temperature (Figure 6b). It was also noted that the final temperature below 38 °C would not influence the morphology of tumor tissue if the nanoaggregates were only injected without PTA process upon pulse laser, which again indicated that CW laser do not triggered delivery (Figure S35, Supporting Information).

During the following 14 days, the digital images, tumor volumes, and body weights of mice in all 5 groups were recorded and analyzed. As is displayed in Figure 6c,e and Figure S36, Supporting Information, the solid tumor on mice in the PTA + PTT group were gradually shrunk and obviously eliminated without any reoccurrence. Nevertheless, there is almost no therapeutic effect in the groups with only 808 m CW or CW plus pulse laser irradiation but without nanoaggregates administered (group 4 as control and group 5). In comparison, despite that the EPR + PTT group exhibited an inhibition effect in the first 4 days, the tumor would still regrow afterward, which can be ascribed to the incomplete killing of cancer cells in barely EPR. The body weight of mice in all groups kept stable within a normal range during the observation (Figure 6d). In addition, more severe signs of necrosis in the PTA + PTT group can be seen according to the haematoxylin and eosin (H&E) staining (Figure 6e). Moreover, the therapeutic process of PTA + PTT only took 40 min of 808 nm pulse laser scanning, while the traditional EPR for even 24 hours before PTT cannot reach the same level of tumor‐killing effectiveness as PTA‐guided delivery. The biosafety of our designed TBT‐2(TP‐DPA) nanoaggregates administered in the mice were also investigated and analysed via blood tests and H&E staining (Figures S37–S39, Supporting Information). All the blood routine and serum biochemistry results show that there was no significant difference between the nanoaggregates treated group at 14 days post injection and the control group. The main organs collected from the mice sacrificed on day 14 post injection showed no obvious lesion or damage, including the heart, liver, spleen, lung, and kidney. These results collectively confirmed the biosafety of TBT‐2(TP‐DPA) nanoaggregates for potential bio‐applications.

This work offers new NIR‐absorbing nanoaggregates based on small molecules, which not only perform promising multifunctional phototheranostics (FLI, PAI, PTI, and PTT) effectiveness but are also adapted to the approach and platform of drug delivery enabled by photoinduced thermoacoustic process. With this strategy, there is a reason to believe that other NIR‐absorbing small molecule‐based nanoaggregates are also promising in this PTA‐guided fast delivery and efficient cancer treatment, which would make up the shortcoming of EPR and save some time for tumor‐targeting design.

## 3 Conclusion

In summary, we developed a multifunctional one‐for‐all NIR‐absorbing small molecule, TBT‐2(TP‐DPA), for cancer phototheranostics. The nanoaggregates of TBT‐2(TP‐DPA), which is simply encapsulated by DSPE‐PEG, can perform photothermal conversion efficiency up to 51%, while remaining an appropriate quantum yield for NIR‐II imaging. The photoacoustic response of the nanoaggregates was also investigated, exhibiting a linear relationship between nanoaggregate concentrations and photoacoustic signals. Furthermore, we developed and investigated a simple physical delivery method, the PTA process, and the results showed that the photoacoustic effect can be used as an imaging technique and a driving force for fast drug delivery in vitro and in vivo. Moreover, the nanoaggregates accumulation in the tumor region can not only be visualized on photoacoustic microscopy, but also triggered by the 808 nm pulse laser in the dual‐wavelength photoacoustic system thanks to the PTA process, as we first reported in this work. Interestingly, the PTA‐driven strategy with laser scanning for 40 min can lead to more accumulation and more effective PTT than barely EPR for 24 h, with complete elimination of cancer cells and no reoccurrence of tumors in a noninvasive and biosafe manner. This simple physical strategy can reduce the toxic side effect of existing phototheranostics nanomedicine since lower doses and milder laser irradiation can be achieved in PTA‐enabled accumulations. Thus, the combination of the NIR‐absorbing nanoaggregates and the PTA‐enabled fast delivery platform reported in this work is feasible and significant to boost the development of efficient one‐for‐all phototheranostics and other clinical applications.

## Conflict of Interest

The authors declare no conflict of interest.

## Supporting information

Supporting Information

## Data Availability

The data that support the findings of this study are available from the corresponding author upon reasonable request.
