# Supplementary material for: Fast Delivery of Multifunctional NIR‐II Theranostic Nanoaggregates Enabled by the Photoinduced Thermoacoustic Process
Source: Adv Sci (Weinh). 2023 Apr 23;10(19):2301104. doi: 10.1002/advs.202301104 (PMC10323613; doi:10.1002/advs.202301104)
Supplement: Supplementary file 1 — Supporting Information [file ADVS-10-2301104-s001.pdf]

ADVANCED SCIENCE
Open Access

Supporting Information

for *Adv. Sci.*, DOI 10.1002/advs.202301104

Fast Delivery of Multifunctional NIR-II Theranostic Nanoaggregates Enabled by the Photoinduced Thermoacoustic Process

Huilin Xie, Chen Zhang, Tingting Li, Lianrui Hu, Jianquan Zhang, Heng Guo, Zhao Liu, Dinglu Peng, Zeshun Li, Weijun Wu, Ji Gao, Zhenyu Bi, Jinghan Wang, Pengfei Zhang, Ryan T. K. Kwok, Jacky W. Y. Lam, Zhihong Guo, Lei Xi, Kai Li* and Ben Zhong Tang**

Supporting Information

Fast Delivery of Multifunctional NIR-II Theranostic Nanoaggregates Enabled by the Photoinduced Thermoacoustic Process

Huilin Xie,[#] Chen Zhang,[#] Tingting Li,[#] Lianrui Hu, Jianquan Zhang, Heng Guo, Zhao Liu, Dinglu Peng, Zeshun Li, Weijun Wu, Ji Gao, Zhenyu Bi, Jinghan Wang, Pengfei Zhang, Ryan T. K. Kwok, Jacky W. Y. Lam, Zhihong Guo, Lei Xi*, Kai Li*, Ben Zhong Tang*

Materials and Methods

Materials

Chemicals and solvents were all commercially available without further purification unless specified. 1,2-Distearoyl-*sn*-glycero-3-phosphoethanolamine-*N*-[methoxy(polyethylene glycol)-2000](DSPE-PEG_{2000}) were purchased from Nanocos (USA). Cell Counting Kit-8 assay, Calcein-AM, and PI were acquired from Beyotime Biotechnology. Roswell Park Memorial Institute (RPMI) 1640, penicillin-streptomycin solution, and trypsin-EDTA (0.5% trypsin and 5.3 mm EDTA tetrasodium) were obtained from Gibco (USA). Milli-Q water (18.2 MΩ cm) was used in all experiments.

Characterization

UV-vis-NIR spectra were measured using Shimadzu (Japan) UV-2600 spectrophotometer. Fluorescence spectra were recorded by Hitachi (Japan) F-4600 fluorescence spectrophotometer. Absolute quantum yield was measured by Hamamatsu UV-NIR Absolute PL quantum yield spectrometer C13534. The size distribution of nanoparticles was determined by dynamic light scattering (DLS) using Malvern (UK) Nano ZS Zetasizer. Morphological structures of the nanoparticles were studied by transmission electron microscopy (TEM) using Hitachi (Japan) TEM-HT7700 at 100 kV accelerating voltage. *In vivo* NIR-II fluorescence imaging results were recorded on a Series III 900/1700-D NIR-II imaging system (Yingrui, Suzhou). *In vivo* photoacoustic imaging was performed by a commercial ORPAM system (NIR-VIS-50, PAOMTek Inc.). Flow cytometry analysis was acquired on a FACSCanto Analyzer. The 808 nm laser was purchased from Changchun Laser Technology Co., Ltd (Changchun, China) and the infrared thermal images were acquired by FLIR E6 thermal imagers. Fluorescence confocal imaging was conducted on a Zeiss LSM980 confocal microscopy.

Preparation of TBT-2(TP-DPA) nanoparticles

The compound of TBT-2(TP-DPA) was first synthesized and purified according to modified reactions. In brief, TBT-2(TP-DPA) (1 mg) and DSPE-PEG_{2000} (1 mg) were dissolved in THF (1 mL) by sonication, then mixed with 9 mL of deionized water. The mixture was sonicated via an ultrasound probe (VCX150, Sonics) for 2 min at 75 W output. THF was removed through dialysis against DI water overnight in a dialysis bag

(molecular weight cut off = 8000-14000 Da). The solution was filtered through a 0.2 μm syringe filter to yield nanoparticles suspended in water for further characterization.

In vitro photoacoustic imaging

Different concentrations (50, 100, 200, 500 μg mL^{-1}) of TBT-2(TP-DPA) PTA nanoaggregates aqueous solution were prepared and sealed in rubber tubes respectively. Then the solutions of the nanoaggregates were imaged using an AR-PAM (acoustic-resolution photoacoustic microscopy) system with an 808 nm laser.

In vivo photoacoustic and NIR-II fluorescence imaging

4T1 tumor-bearing mice were intravenously injected with TBT-2(TP-DPA) PTA nanoaggregates (200 μL, 1 mg mL^{-1}). The mice were placed on the imaging end of a commercial ORPAM system (NIR-VIS-50, PAOMTek Inc.), which provides a field of view (FOV) of 10 mm, and lateral resolutions of 10.4 μm for 532 nm and 13.1 μm for 808 nm, respectively. Each scan takes round 20 s. The TBT-2(TP-DPA) nanoaggregates and solid tumors were scanned with an 808 nm pulse laser at 200 mW (61 mJ cm^{-2}) power for 30 min. For the PTA group, the photoacoustic signal of the tumor was monitored after each scan. The mice were subsequently irradiated by a diverged high-energy 808 nm pulse laser (27.5 mJ single-pulse energy) equipped on the PAT system for another 10 min. After scanning, the tumor was imaged by the NIR-II fluorescence imaging system. For the EPR group, the NIR-II fluorescence imaging was directly obtained at different time points without photoacoustic imaging.

In vitro photothermal measurement

To test the *in vitro* concentration-dependent photothermal effect, 200 μL of TBT-2(TP-DPA) PTA nanoaggregates solution with different concentrations (0.03125, 0.0625, 0.125, 0.25, 0.5 and 1 mg mL^{-1}) was added into 96 well cell culture plate with 808 nm laser (1 W cm^{-2}) for 5 min. To test the *in vitro* laser energy-dependent photothermal effect, 200 μL of TBT-2(TP-DPA) PTA nanoaggregates solution at 1 mg mL^{-1} was added into 96 well cell culture plate with 808 nm laser with different influence (0.2, 0.4, 0.6, 0.8 and 1 W cm^{-2}) for 5 min. 200 μL of deionized water was also irradiated under the same condition as the control group. IR thermal camera was used to image the temperature changes. To test the photothermal stability of TBT-2(TP-DPA) PTA nanoaggregates, five cycles of heating and cooling were recorded.

In Vitro Cytotoxicity

4T1 cells were seeded into a 96-well plate at a density of 1 × 10^4 cells and cultured at 5% CO_2 and 37 °C for 24 h. Following, the initial medium was replaced with a fresh medium containing different concentrations (0, 10, 25, 50, 100, and 250 μg mL^{-1}) of TBT-2(TP-DPA) PTA nanoaggregates to incubate the cells for another 24 h. Next, the culture media was replaced by fresh media containing 10% CCK-8 medium solution and cultured for 2 h. The cell viability was calculated by measuring the absorbance value on a microplate reader at 450 nm.

Live and Dead Cell Assay

4T1 cells were seeded into the confocal dish at a density of 1 × 10^5 cells and cultured at 5% CO_2 and 37 °C for 12 h. TBT-2(TP-DPA) PTA nanoaggregates (0.05 mg mL^{-1}, 200 μL) were then added into the cell culture medium. After incubation of 12 h, the cells were washed and the culture media was replaced by fresh media. For PTT *in vitro*, the treatment groups were irradiated with 808 nm CW laser (1 W cm^{-2}) for 10 min. Then, the medium was removed and washed with 1 × PBS twice. The cells were successively incubated with Calcein-AM (100 μL, 5 μM) at 37 °C for 15 min and PI solution (100 μL, 50 μM) at room temperature for 15 min. The cells were gently washed and imaged by confocal microscopy.

Flow Cytometry Analysis

4T1 cells were seeded into 12-well microplates at a density of 1 × 10^5 cells and cultured at 5% CO_2 and 37 °C for 12 h. TBT-2(TP-DPA) PTA nanoaggregates (1 mg mL^{-1}, 200 μL) were added into the cell culture plate and incubated with 4T1 cells for 12 h. For PTT, the treatment groups were irradiated with 808 nm CW laser (1 W cm^{-2}) for 10 min. The supernatant was collected, and the cells were digested by 0.25% trypsin-EDTA solution and stained with annexin V-FITC/PI (Sangon Biotech), followed by analysis on FACSCanto Analyzer.

In Vivo Biosafety Analysis

The female BALB/c mice (4–6 weeks, ~20 g) were purchased from the Guangdong Medical Laboratory Animal Center. The animal procedures were approved by the Institutional Animal Care and Use Committee of Southern University of Science and

Technology. The mice were treated with the TBT-2(TP-DPA) PTA nanoaggregates (1 mg mL^{-1}, 200 μL) through tail vein injection. The control group was injected with 1 × PBS buffer at the same volume. Before the mice were sacrificed, the blood was collected for into blood collection tubes immediately by enucleation of mouse eyes for haematology analysis. For biochemistry analysis, the rest of the blood sample was kept at room temperature for 2 h and then centrifuged at 4000 rpm for 10 min to collect the supernatant serum for use. The main organs (heart, liver, spleen, lung, and kidney) were collected at 0-, 7-, and 14-days post-injection, and were stained with H&E for histological analysis.

In Vivo Photothermal Therapy

4T1 cells (2 × 10^6 cells in 1 × PBS buffer) were injected subcutaneously into the flank of the female BALB/c nude mice (4 weeks). When the tumor volume reached 80 mm^3, the mice were randomly divided into 5 groups (n = 3 each group) and given the following treatments: 1) TBT-2(TP-DPA) nanoaggregates + 808 nm pulse laser + 808 nm CW laser (PTA 40 min + PTT); 2) TBT-2(TP-DPA) nanoaggregates + 808 nm CW laser (EPR 24 h + PTT); 3) TBT-2(TP-DPA) nanoaggregates + 808 nm pulse laser (PTA); 4) 808 nm pulse laser + 808 nm CW laser; and 5) PBS, respectively. All the nanoaggregates were injected at the concentration of 1 mg mL-1 for a volume of 200 μL. For the PTA group, the tumors were irradiated by an 808 nm pulse laser for 40 min after injection of TBT-2(TP-DPA) PTA nanoaggregates. For PTA + PTT group, the tumors were irradiated by an 808 nm pulse laser for 40 min immediately after injection of TBT-2(TP-DPA) nanoaggregates, followed by irradiation of 808 nm CW laser (1 W cm^{-2}) for 10 min. For EPR 24 h + PTT group, the tumors were directly irradiated by an 808 nm CW laser (1 W cm^{-2}) for 10 min at 24 h post injection. For the PTA group, the tumors were only irradiated by an 808 nm pulse laser for 40 min without 808 nm CW laser irradiation, immediately post injection. The tumor volume was measured by a digital caliper every 2 days. The tumor volume = length × $width^2/2$.

Statistical information

All results were expressed as mean ± standard deviation through at least three experiments. One-way analysis of variance (ANOVA) was used for the statistical analysis of data between 2 groups. $p < 0.05$ was considered statistically significant. 0.01

$< *P < 0.05$, $0.001 < **P < 0.01$, $***P < 0.001$. All the statistical calculations were conducted by Origin 2021b software.

Supplementary Figures and Tables

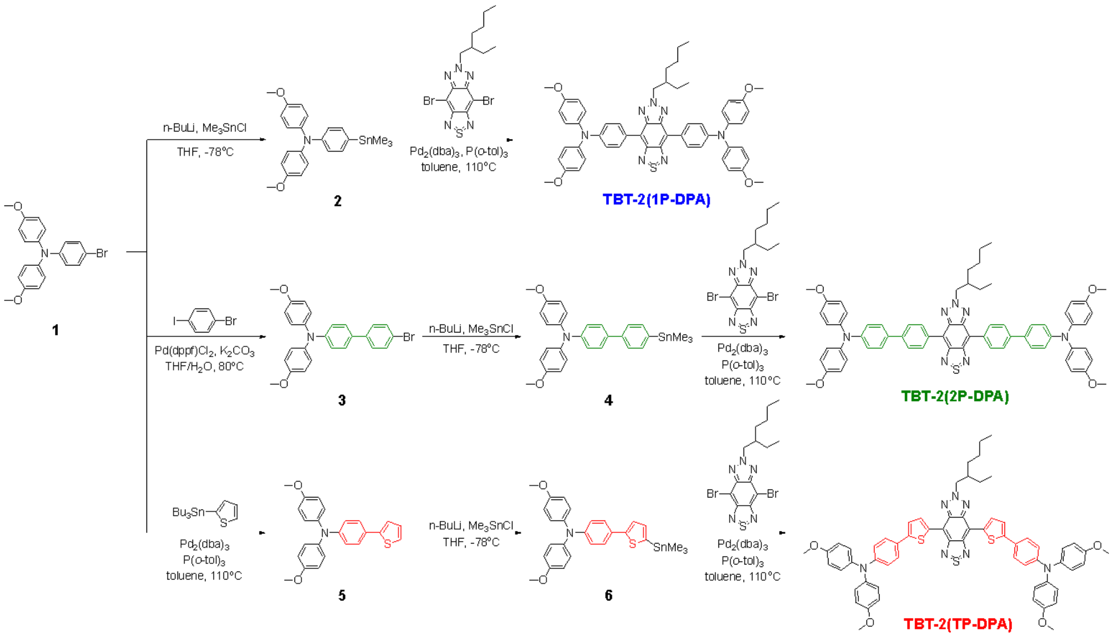

Scheme S1. Synthetic route to **TBT-2(1P-DPA), TBT-2(2P-DPA), and TBT-2(TP-DPA)**.

Synthesis of 4-methoxy-N-(4-methoxyphenyl)-N-(4-(trimethylstannyl)phenyl)-aniline (2).

To a solution of 4-bromo-N,N-bis(4-methoxyphenyl)aniline (**1**, 1.00 g, 2.60 mmol) in anhydrous THF (30 mL) was added *n*-BuLi (1.3 mL, 2.5 M in hexane) at -78°C under N_2 atmosphere. The mixture was stirred at the same temperature for one hour, followed by the addition of trimethyltin chloride (3.4 mL, 3.38 mmol, 1.0 M in hexane). The reaction was allowed to slowly warm up to room temperaure and stirred overnight. The reaction mixture was quenched by aqueous KF solution, wahsed with water and brine, and dried over Na_2SO_4. The crude product was obtained by concentration under reduced pressure and used without futher purification.

Synthesis of TBT-2(1P-DPA).

A mixture of 4,8-dibromo-6-(2-ethylhexyl)[1,2,5]thiadiazolo[3,4-f]benzotriazole (100 mg, 0.22 mmol), compound **2** (314 mg, 0.67 mmol), $Pd_2(dba)_3$ (20 mg, 0.022 mmol), and P(*o*-tol)$_3$ (54 mg, 0.18 mmol) were dissolved in anhydrous toluene (10 mL) and

stirred at 110 °C overnight under N_2 atmosphere. After being cooled to room temperature, the reaction mixture was extracted with chloroform, washed with aqueous KF solution, water and brine. After concentration under reduced pressure, the crude product was purified by column chromatography (stationary phase: silica gel; eluent: dichloromethane) to get the product as a blue solid (150 mg, 75%). ^{1}H NMR (400 MHz, $CDCl_3$) δ 8.36 (d, *J* = 8.7 Hz, 4H), 7.26 – 7.18 (m, 8H), 7.15 (d, *J* = 8.5 Hz, 4H), 6.95 – 6.85 (m, 8H), 4.81 (d, *J* = 7.0 Hz, 2H), 3.85 (s, 12H), 2.39 (q, *J* = 6.3 Hz, 1H), 1.48 – 1.24 (m, 8H), 0.99 (t, *J* = 7.4 Hz, 3H), 0.89 (t, *J* = 7.2 Hz, 3H). ^{13}C NMR (101 MHz, $CDCl_3$) δ 156.26, 151.49, 148.67, 144.37, 140.48, 132.01, 127.31, 126.82, 119.21, 114.77, 61.20, 55.53, 40.54, 30.63, 28.43, 24.06, 23.00, 14.07, 10.57. MS (ESI) m/z calcd. for $C_{54}H_{53}N_7O_4S^+$: 895.3880. Found: 895.3883.

Synthesis of 4'-bromo-N,N-bis(4-methoxyphenyl)-[1,1'-biphenyl]-4-amine (3).

A mixture of compound **1** (1.00 g, 2.60 mmol), 4-bromophenylboronic acid (522 g, 2.60 mmol), Pd(dppf)Cl_2 (106 mg, 0.13 mmol) and K_2CO_3 (1.44 g, 10.42 mmol) were dissolved in THF/water (20/10 mL) and stirred at 80 °C overnight under N_2 atmosphere. After being cooled to room temperature, the reaction mixture was extracted with chloroform, washed with water and brine. After concentration under reduced pressure, the crude product was purified by column chromatography (stationary phase: silica gel; eluent: dichloromethane) to get the product as a white solid (741 mg, 62%). ^{1}H NMR (400 MHz, $CDCl_3$) δ 7.53 (d, *J* = 8.4 Hz, 2H), 7.43 (d, *J* = 8.6 Hz, 2H), 7.39 (d, *J* = 8.6 Hz, 2H), 7.11 (d, *J* = 8.9 Hz, 4H), 7.00 (d, *J* = 8.7 Hz, 2H), 6.87 (d, *J* = 8.9 Hz, 4H), 3.83 (s, 6H). ^{13}C NMR (101 MHz, $CDCl_3$) δ 156.02, 148.49, 140.71, 139.76, 131.76, 131.55, 128.02, 127.33, 126.75, 120.50, 114.76, 55.51.

Synthesis of N,N-bis(4-methoxyphenyl)-4'-(trimethylstannyl)-[1,1'-biphenyl]-4-amine (4).

To a solution of compound **5** (600 mg, 1.09 mmol) in anhydrous THF (10 mL) was added *n*-BuLi (0.5 mL, 2.5 M in hexane) at -78°C under N_2 atmosphere. The mixture was stirred at the same temperature for one hour, followed by the addition of trimethyltin chloride (1.4 mL, 1.42 mmol, 1.0 M in hexane). The reaction was allowed to slowly warm up to room temperaure and stirred overnight. The reaction mixture was

quenched by aqueous KF solution, wahsed with water and brine, and dried over Na_2SO_4. The crude product was obtained by concentration under reduced pressure and used without futher purification.

Synthesis of TBT-2(2P-DPA).

A mixture of 4,8-dibromo-6-(2-ethylhexyl)[1,2,5]thiadiazolo[3,4-f]benzotriazole (100 mg, 0.22 mmol), compound **6** (365 mg, 0.67 mmol), $Pd_2(dba)_3$ (20 mg, 0.022 mmol), and P(*o*-tol)$_3$ (54 mg, 0.18 mmol) were dissolved in anhydrous toluene (10 mL) and stirred at 110 °C overnight under N_2 atmosphere. After being cooled to room temperature, the reaction mixture was extracted with chloroform, washed with aqueous KF solution, water and brine. After concentration under reduced pressure, the crude product was purified by column chromatography (stationary phase: silica gel; eluent: dichloromethane) to get the product as a blue solid (192 mg, 82%). ^{1}H NMR (400 MHz, $CDCl_3$) δ 8.51 (d, *J* = 8.4 Hz, 4H), 7.84 (d, *J* = 8.5 Hz, 4H), 7.60 (d, *J* = 8.7 Hz, 4H), 7.17 (d, *J* = 8.9 Hz, 8H), 7.07 (d, *J* = 8.7 Hz, 4H), 6.90 (d, *J* = 8.9 Hz, 8H), 4.88 (d, *J* = 7.0 Hz, 2H), 3.85 (s, 12H), 2.47 (q, *J* = 6.4 Hz, 1H), 1.53 – 1.32 (m, 8H), 1.04 (t, *J* = 7.4 Hz, 3H), 0.95 (t, *J* = 7.0 Hz, 3H). ^{13}C NMR (101 MHz, $CDCl_3$) δ 156.01, 151.61, 148.41, 144.68, 140.83, 133.16, 132.45, 131.70, 127.60, 126.81, 126.41, 120.50, 118.53, 114.78, 61.42, 55.53, 40.58, 30.61, 28.38, 24.08, 23.02, 14.10, 10.60. MS (ESI) m/z calcd. for $C_{66}H_{61}N_7O_4S^+$: 1047.4506. Found: 1047.4507.

Synthesis of 4-methoxy-N-(4-methoxyphenyl)-N-(4-(thiophen-2-yl)phenyl)aniline (5).

A mixture of compound **1** (1.00 g, 2.60 mmol), 2-(tributylstannyl)thiophene (1.17 g, 3.13 mmol), $Pd_2(dba)_3$ (119 mg, 0.13 mmol), and P(*o*-tol)$_3$ (317 mg, 1.04 mmol) were dissolved in anhydrous toluene (10 mL) and stirred at 110 °C overnight under N_2 atmosphere. After being cooled to room temperature, the reaction mixture was extracted with chloroform, washed with aqueous KF solution, water and brine. After concentration under reduced pressure, the crude product was purified by column chromatography (stationary phase: silica gel; eluent: dichloromethane) to get the product as a light yellow oil (745 mg, 74%). ^{1}H NMR (400 MHz, $CDCl_3$) δ 7.50 (d, *J* = 8.7 Hz, 2H), 7.24 (t, *J* = 4.5 Hz, 2H), 7.16 (d, *J* = 8.9 Hz, 4H), 7.09 (dd, *J* = 5.1, 3.6

Hz, 1H), 7.03 (d, *J* = 8.7 Hz, 2H), 6.92 (d, *J* = 9.0 Hz, 4H), 3.85 (s, 6H). ^{13}C NMR (101 MHz, $CDCl_3$) δ 155.41, 147.65, 144.07, 140.17, 127.43, 126.08, 126.05, 122.99, 121.19, 120.12, 114.22, 54.90.

Synthesis of 4-methoxy-N-(4-methoxyphenyl)-N-(4-(5-(trimethylstannyl) thiophen-2-yl)phenyl)aniline (6).

To a solution of compound **7** (600 mg, 1.55 mmol) in anhydrous THF (20 mL) was added *n*-BuLi (0.74 mL, 2.5 M in hexane) at -78°C under N_2 atmosphere. The mixture was stirred at the same temperature for one hour, followed by the addition of trimethyltin chloride (2.01 mL, 2.01 mmol, 1.0 M in hexane). The reaction was allowed to slowly warm up to room temperaure and stirred overnight. The reaction mixture was quenched by aqueous KF solution, wahsed with water and brine, and dried over Na_2SO_4. The crude product was obtained by concentration under reduced pressure and used without futher purification.

Synthesis of TBT-2(TP-DPA).

A mixture of 4,8-dibromo-6-(2-ethylhexyl)[1,2,5]thiadiazolo[3,4-f]benzotriazole (100 mg, 0.22 mmol), compound **8** (370 mg, 0.67 mmol), $Pd_2(dba)_3$ (20 mg, 0.022 mmol), and P(*o*-tol)$_3$ (54 mg, 0.18 mmol) were dissolved in anhydrous toluene (10 mL) and stirred at 110 °C overnight under N_2 atmosphere. After being cooled to room temperature, the reaction mixture was extracted with chloroform, washed with aqueous KF solution, water and brine. After concentration under reduced pressure, the crude product was purified by column chromatography (stationary phase: silica gel; eluent: dichloromethane) to get the product as a blue solid (187 mg, 79%). ^{1}H NMR (400 MHz, $CDCl_3$) δ 8.69 (d, *J* = 4.1 Hz, 2H), 7.57 (d, *J* = 8.7 Hz, 4H), 7.33 (d, *J* = 4.1 Hz, 2H), 7.11 (d, *J* = 8.9 Hz, 8H), 6.96 (d, *J* = 8.8 Hz, 4H), 6.87 (d, *J* = 8.9 Hz, 8H), 4.80 (d, *J* = 6.6 Hz, 2H), 3.82 (s, 12H), 2.34 (p, *J* = 6.2 Hz, 1H), 1.51 – 1.30 (m, 8H), 1.03 (t, *J* = 7.4 Hz, 3H), 0.91 (t, *J* = 7.2 Hz, 3H). ^{13}C NMR (101 MHz, $CDCl_3$) δ 156.07, 149.86, 148.43, 147.90, 142.42, 140.61, 135.85, 132.21, 126.76, 126.63, 126.50, 122.71, 120.41, 114.78, 111.36, 60.69, 55.53, 40.61, 30.72, 28.54, 24.13, 23.00, 14.15, 10.68. MS (ESI) m/z calcd. for $C_{62}H_{57}N_7O_4S_3{}^+$: 1059.3634. Found: 1059.3639.

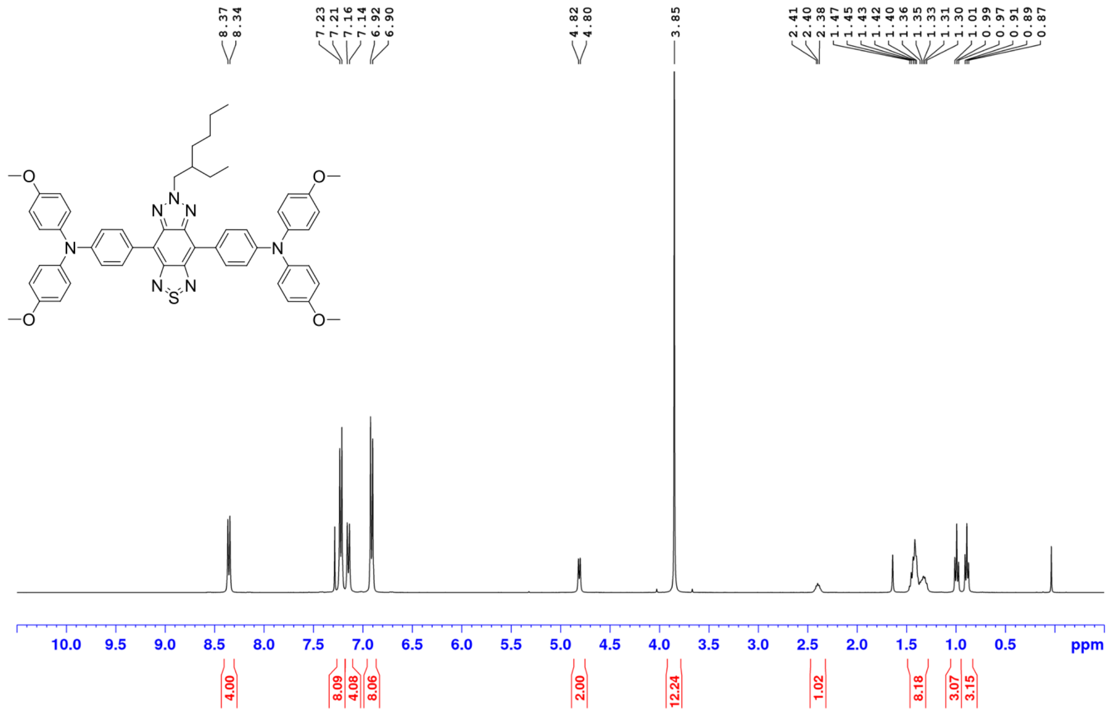

Figure S1. ^{1}H NMR spectrum ($CDCl_3$, 400 MHz, 298 K) of **TBT-2(1P-DPA)**.

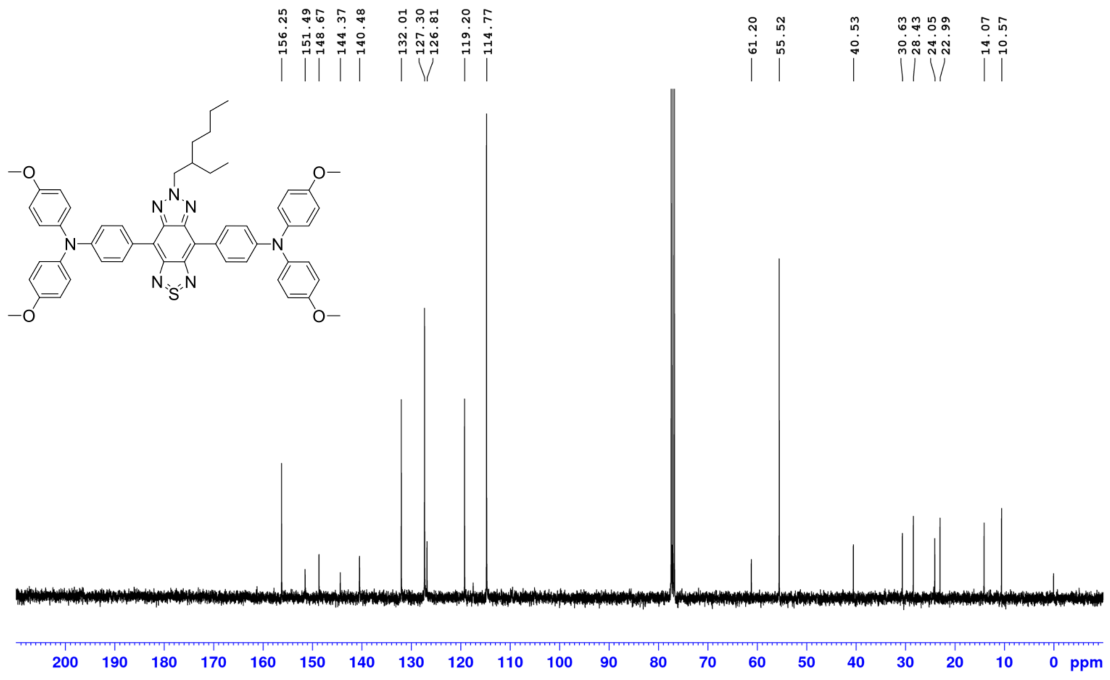

Figure S2. ^{13}C NMR spectrum ($CDCl_3$, 100 MHz, 298 K) of **TBT-2(1P-DPA)**.

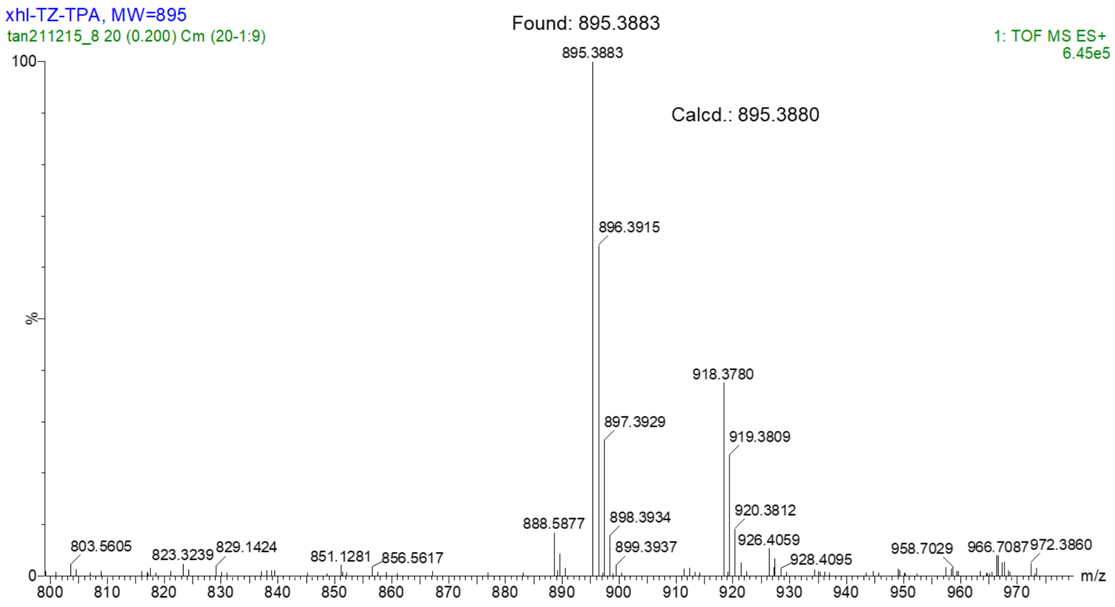

Figure S3. MALDI-TOF mass spectrum of **TBT-2(1P-DPA)**.

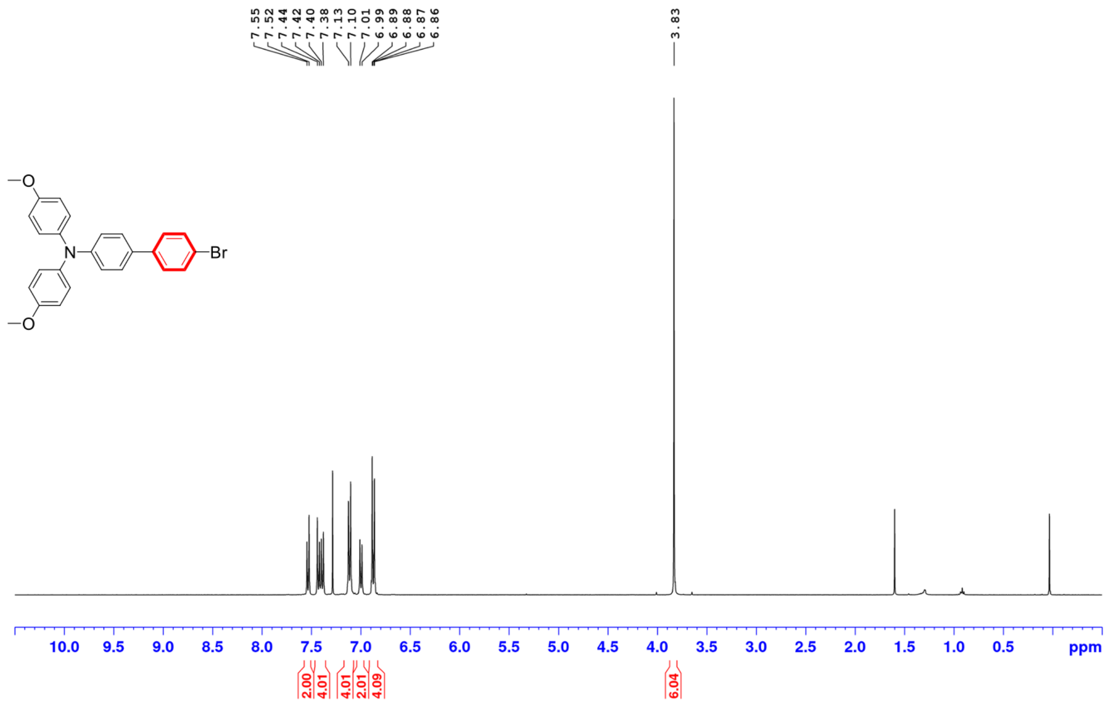

Figure S4. ^{1}H NMR spectrum ($CDCl_3$, 400 MHz, 298 K) of **3**.

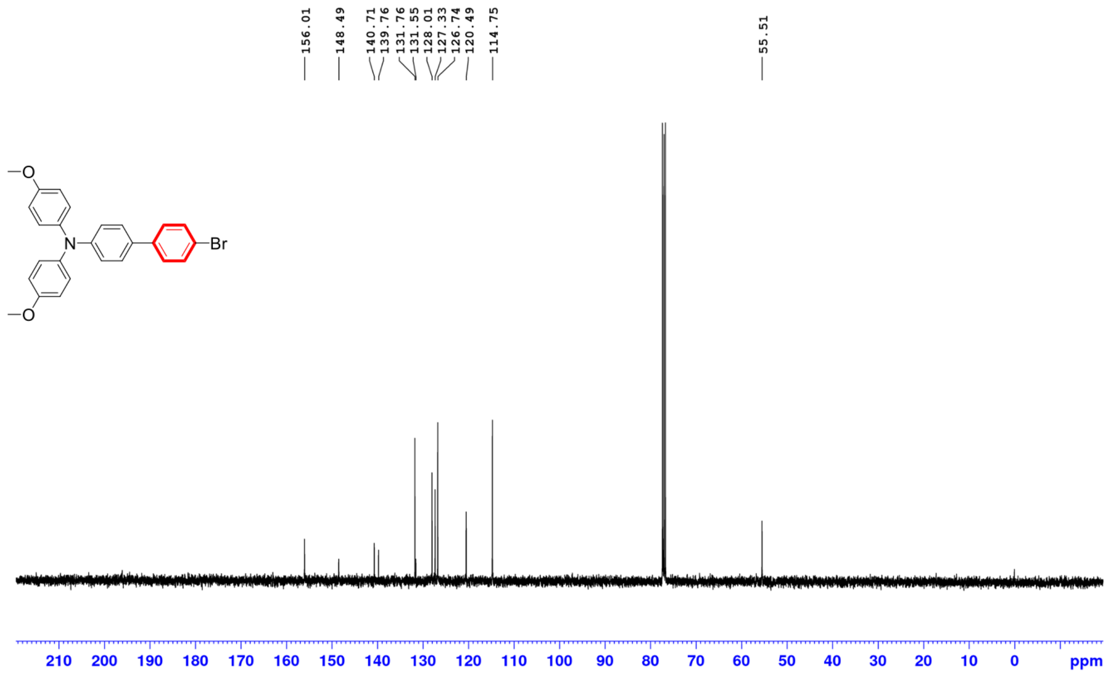

Figure S5. ^{13}C NMR spectrum ($CDCl_3$, 100 MHz, 298 K) of **3**.

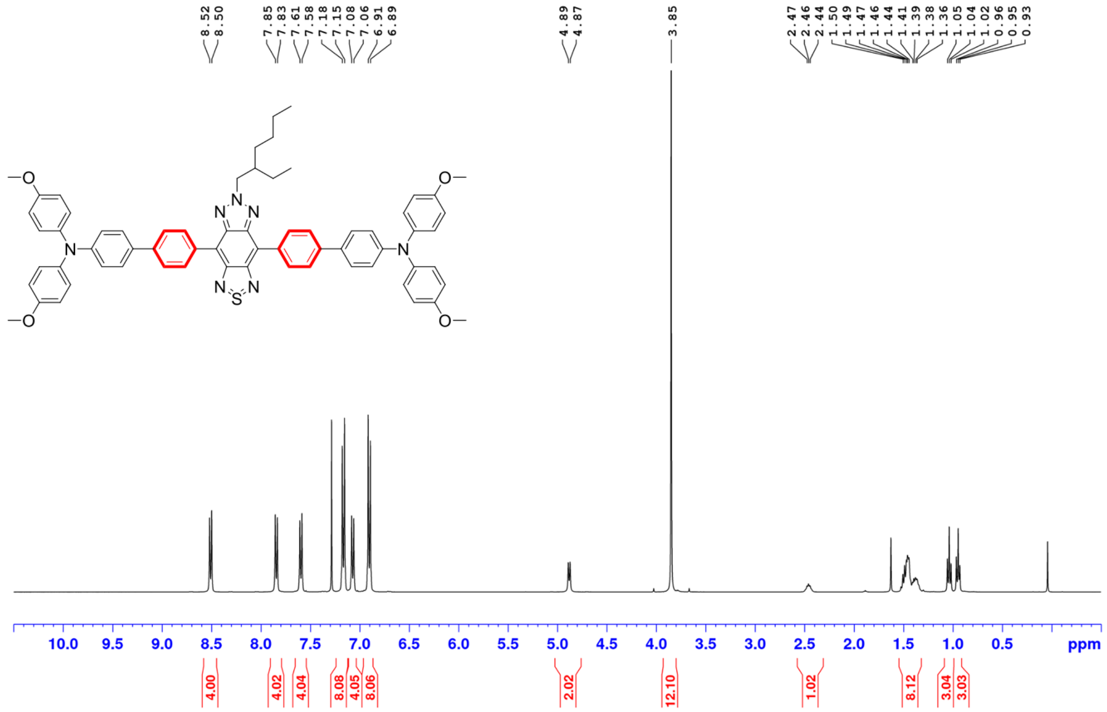

Figure S6. ^{1}H NMR spectrum ($CDCl_3$, 400 MHz, 298 K) of **TBT-2(2P-DPA)**.

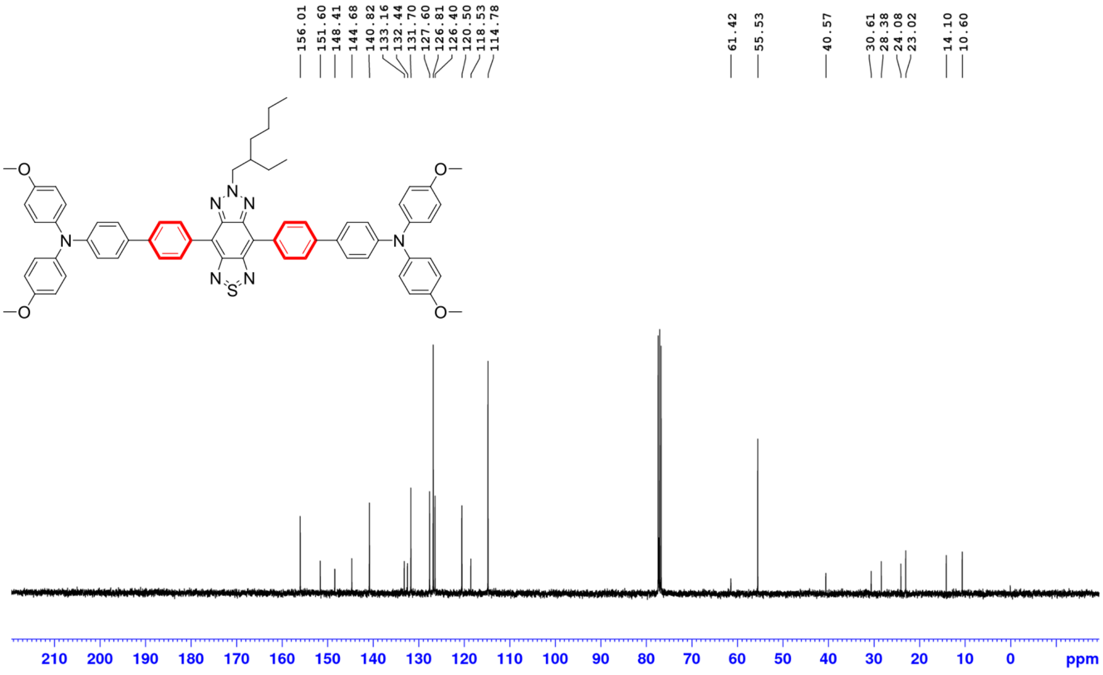

Figure S7. ^{13}C NMR spectrum ($CDCl_3$, 100 MHz, 298 K) of **TBT-2(2P-DPA)**.

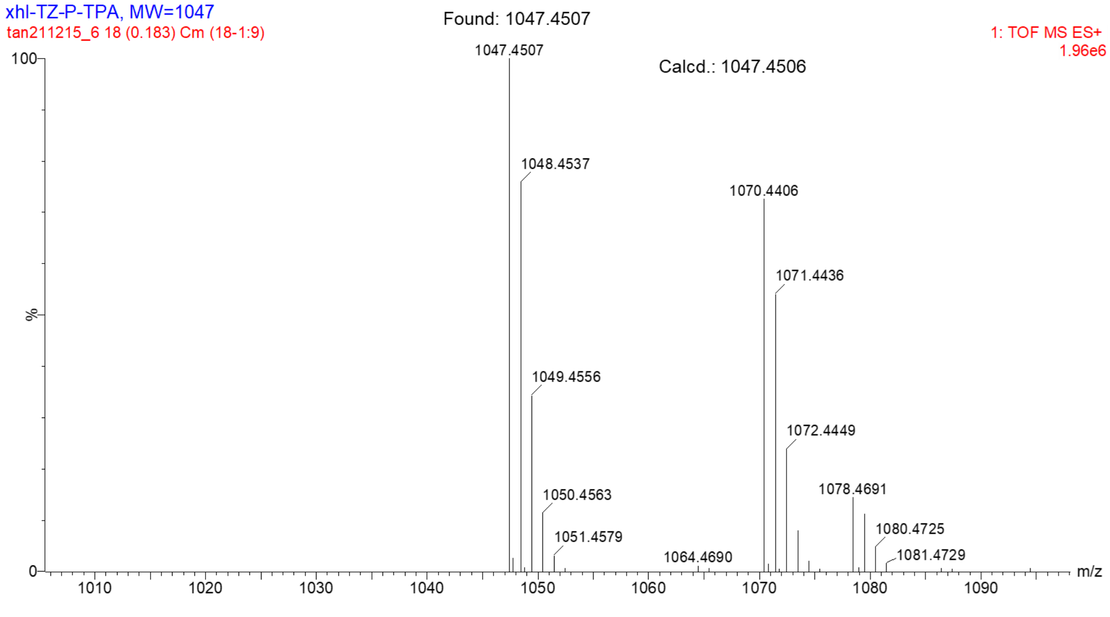

Figure S8. MALDI-TOF mass spectrum of **TBT-2(2P-DPA)**.

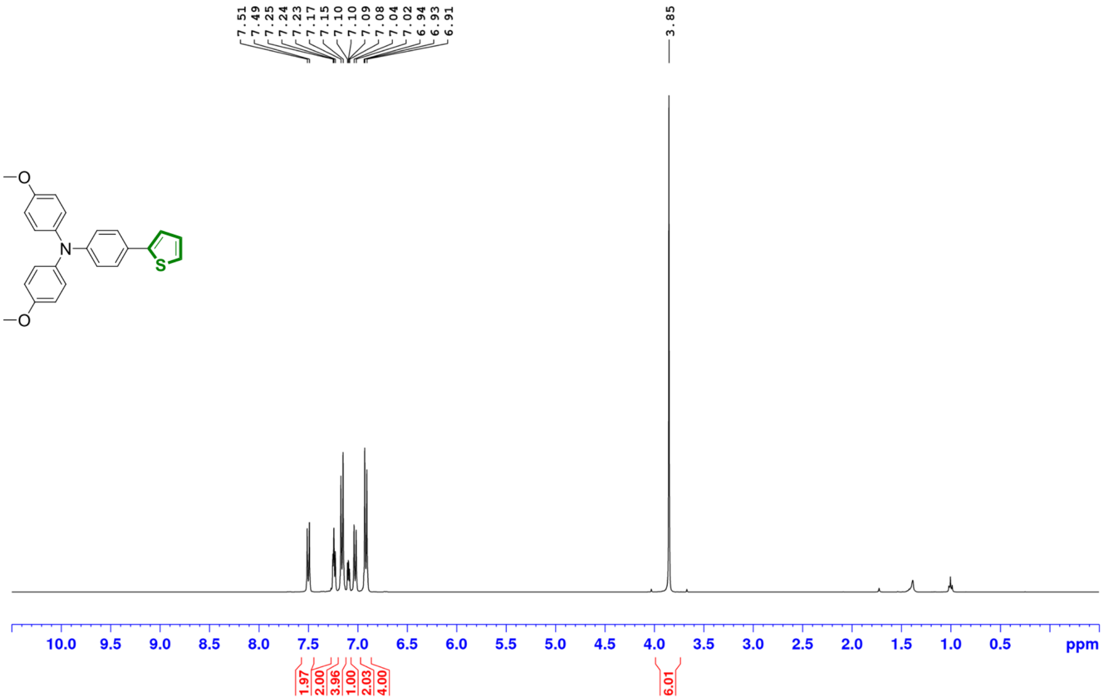

Figure S9. ^{1}H NMR spectrum ($CDCl_3$, 400 MHz, 298 K) of **5**.

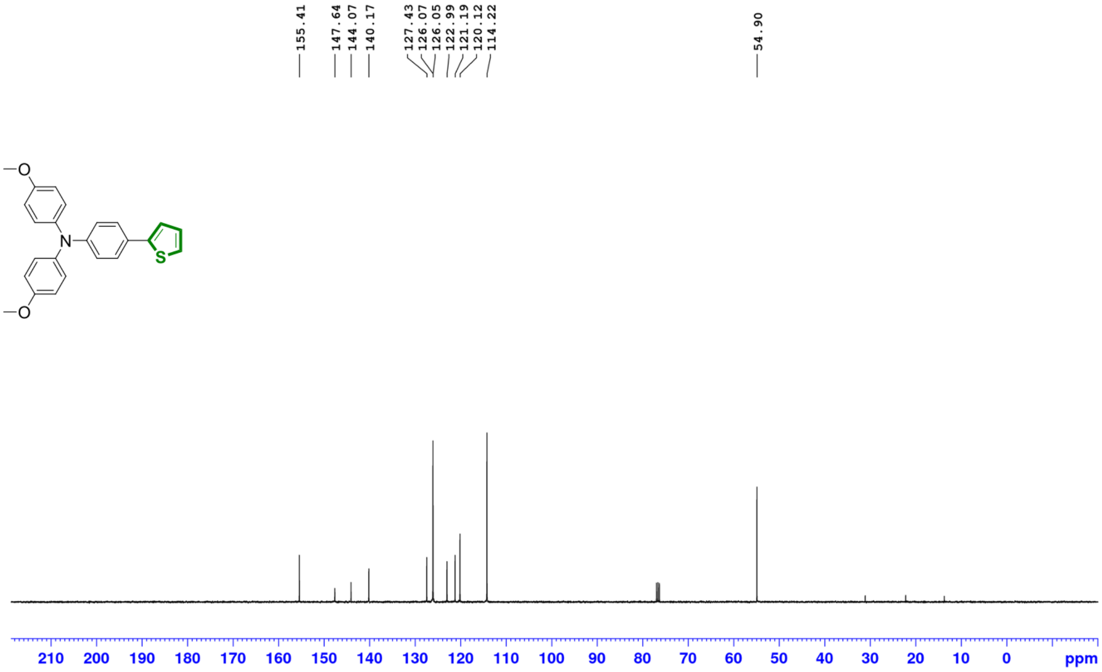

Figure S10. ^{13}C NMR spectrum ($CDCl_3$, 100 MHz, 298 K) of **5**.

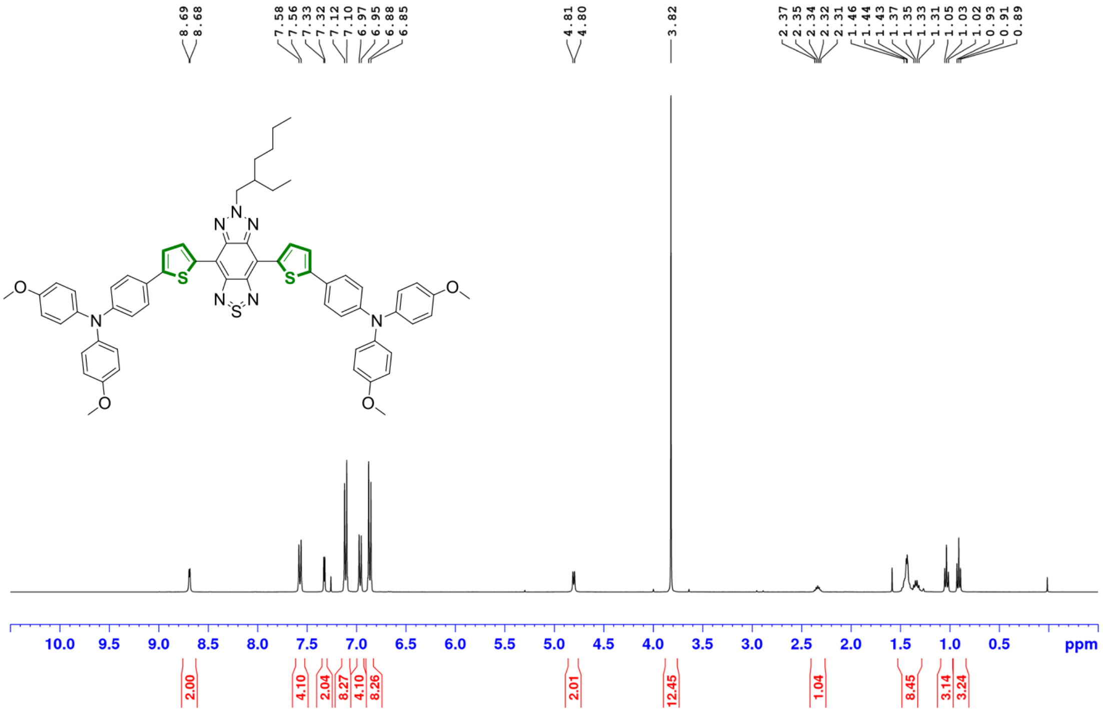

Figure S11. ^{1}H NMR spectrum ($CDCl_3$, 400 MHz, 298 K) of **TBT-2(TP-DPA)**.

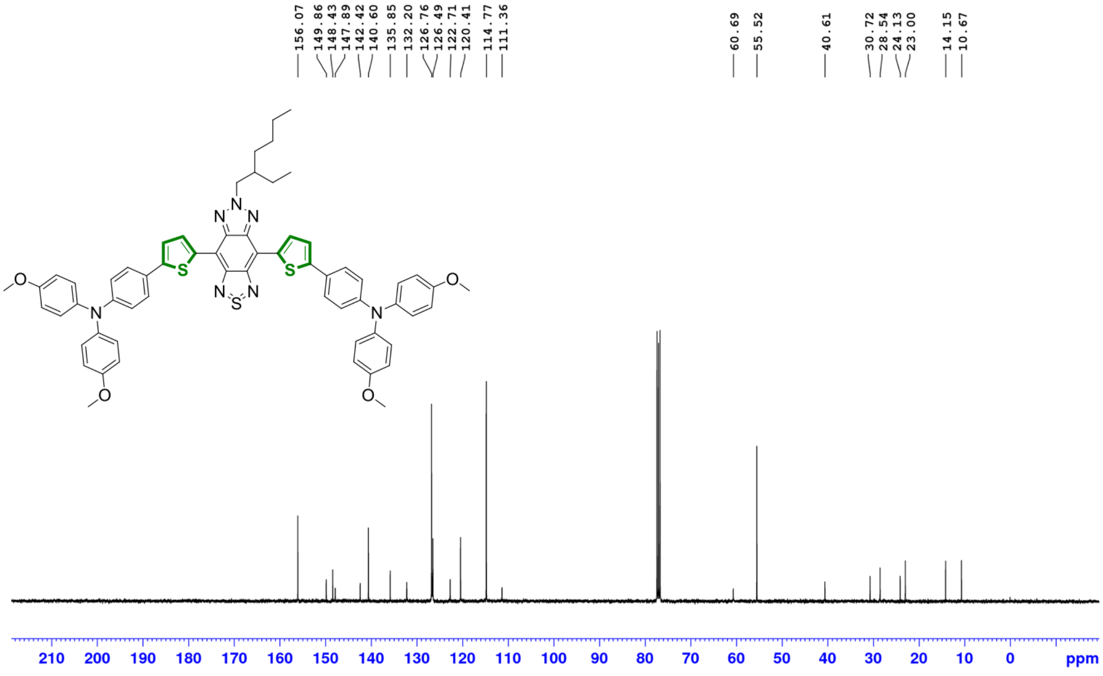

Figure S12. ^{13}C NMR spectrum ($CDCl_3$, 100 MHz, 298 K) of **TBT-2(TP-DPA)**.

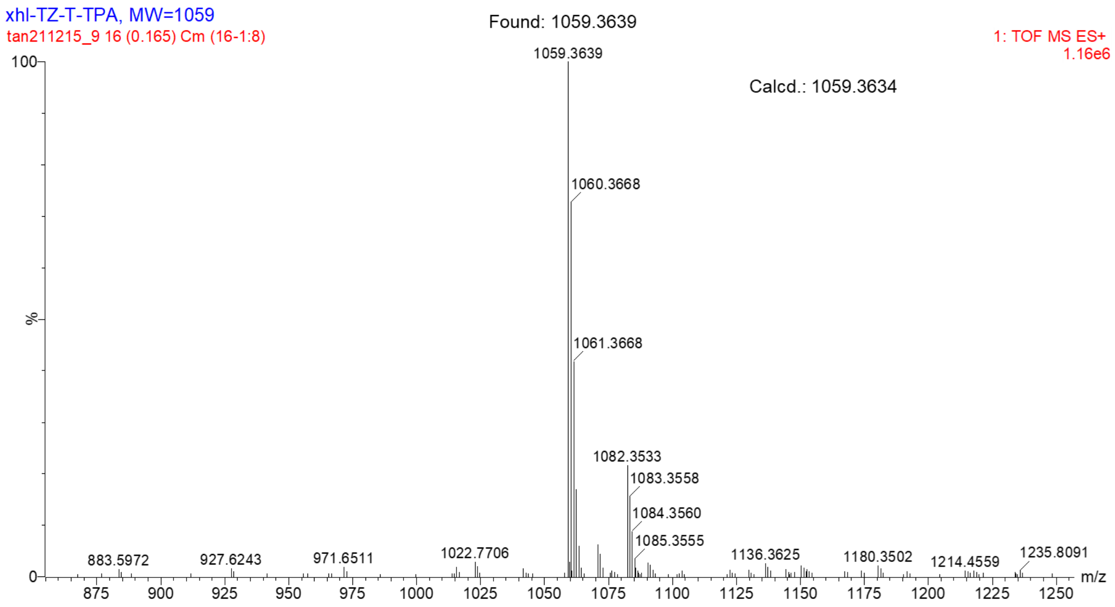

Figure S13. MALDI-TOF mass spectrum of **TBT-2(TP-DPA)**.

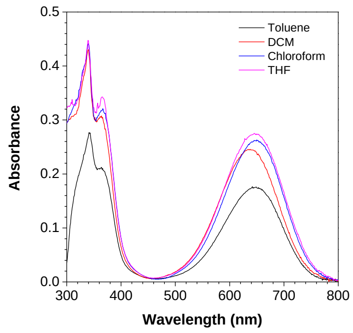

Figure S14. UV-vis spectra of **TBT-2(1P-DPA)** (10^{-5} M) in different solvents.

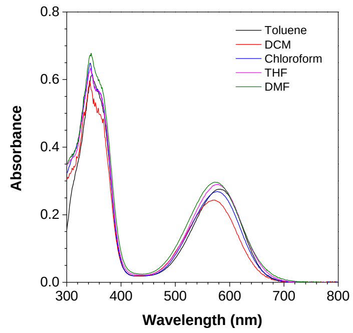

Figure S15. UV-vis-NIR spectra of **TBT-2(2P-DPA)** (10^{-5} M) in different solvents.

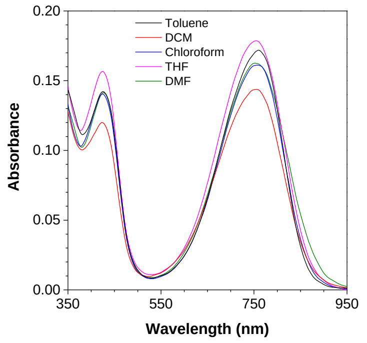

Figure S16. UV-vis spectra of **TBT-2(TP-DPA)** (10^{-5} M) in different solvents.

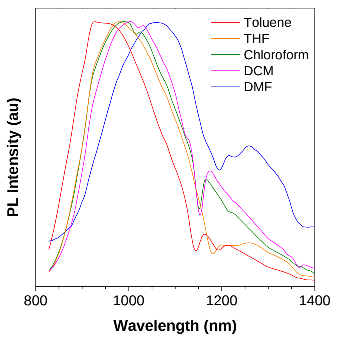

Figure S17. PL spectra of **TBT-2(TP-DPA)** (10^{-5} M) in different solvents.

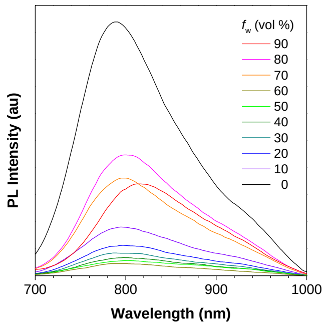

Figure S18. PL spectra of **TBT-2(1P-DPA)** (10^{-5} M) in THF/H_2O mixture with different water fractions (f_w).

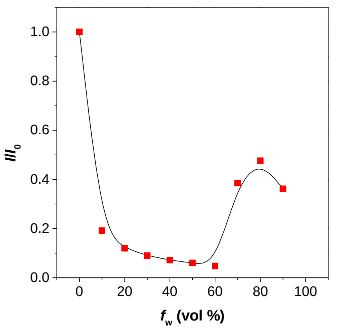

Figure S19. Change of relative PL intensity (I/I_0) of TBT-2(1P-DPA) in THF/H_2O mixture with different f_w, where I = maximum PL intensity at a specific f_w and I_0 = maximum PL intensity at f_w of 0.

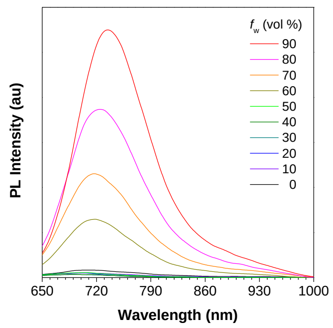

Figure S20. PL spectra of **TBT-2(2P-DPA)** (10^{-5} M) in THF/H_2O mixture with different water fractions (f_w).

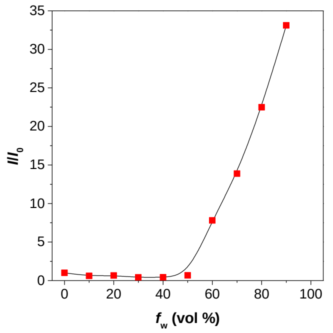

Figure S21. Change of relative PL intensity (I/I_0) of **TBT-2(2P-DPA)** in THF/H_2O mixture with different f_w, where I = maximum PL intensity at a specific f_w and I_0 = maximum PL intensity at f_w of 0.

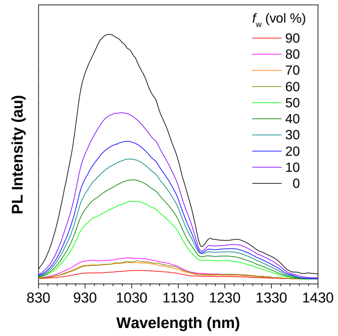

Figure S22. PL spectra of **TBT-2(TP-DPA)** (10^{-5} M) in THF/H_2O mixture with different water fractions (f_w).

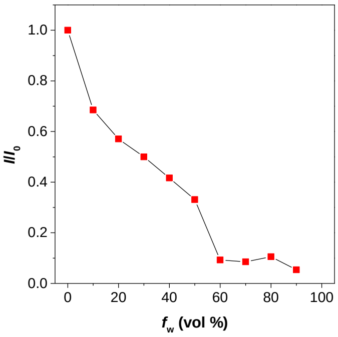

Figure S23. Change of relative PL intensity (I/I_0) of **TBT-2(TP-DPA)** in THF/H_2O mixture with different f_w, where I = maximum PL intensity at a specific f_w and I_0 = maximum PL intensity at f_w of 0.

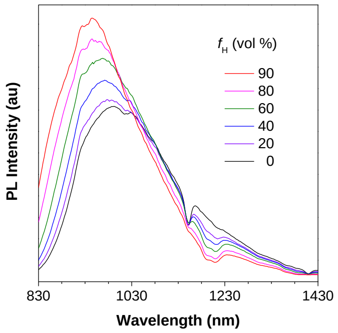

Figure S24. PL spectra of **TBT-2(TP-DPA)** (10^{-5} M) in chloroform/hexane mixture with different hexane fractions (f_H).

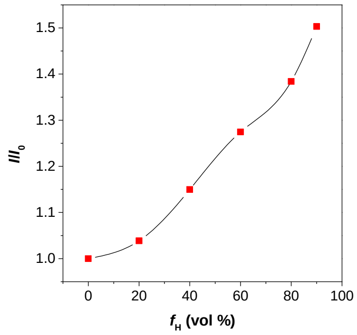

Figure S25. Change of relative PL intensity (I/I_0) of **TBT-2(TP-DPA)** in chloroform/hexane mixture with different f_H, where I = maximum PL intensity at a specific f_H and I_0 = maximum PL intensity at f_H of 0.

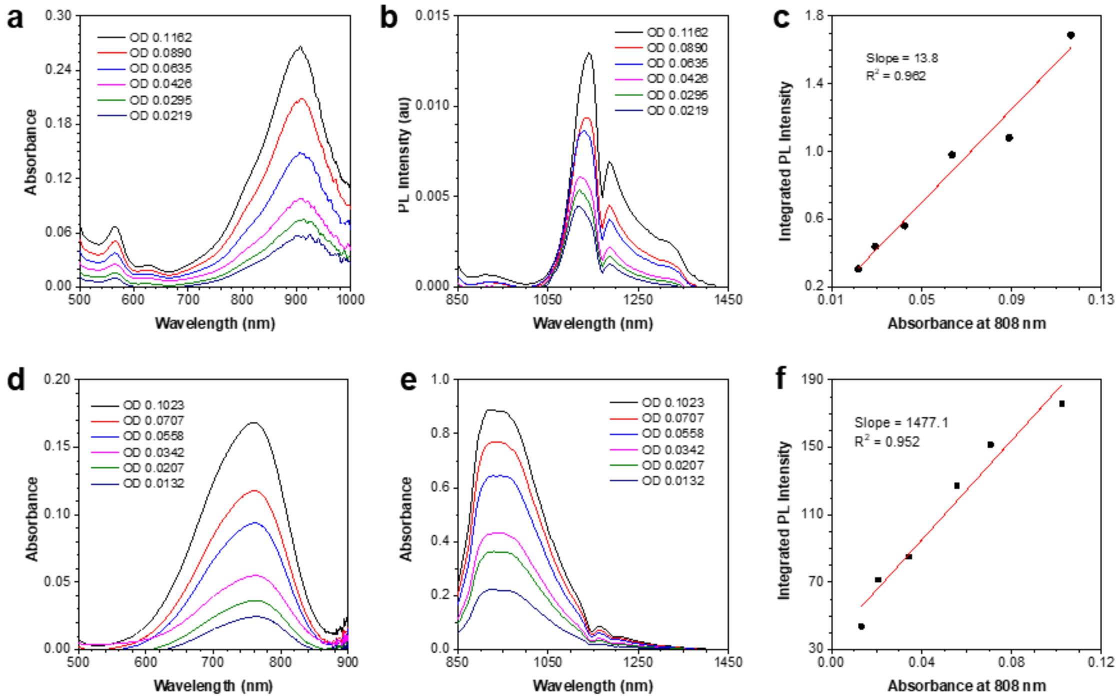

Figure S26. Data in fluorescence quantum yield calculation. UV-Vis-NIR absorption spectra, PL spectra, and a plot of integrated PL intensity vs. the absorbance at 808 nm of IR 26 (a, b, and c) in DCE, and TBT-2(TP-DPA) (d, e, and f) in toluene solution. The quantum yield was calculated in the following manner:

$$QY_{sample} = QY_{IR26} \frac{Slope_{sample}}{Slope_{IR26}} \frac{{n_{sample}}^2}{{n_{IR26}}^2}$$

Where QY_{sample} is the QY of fluorophore TBT-2(TP-DPA) in toluene, QY_{IR26} is the QY

of IR-26 in DCE, n_{sample} and n_{IR26} are the refractive indices of corresponding solvents and DCE.

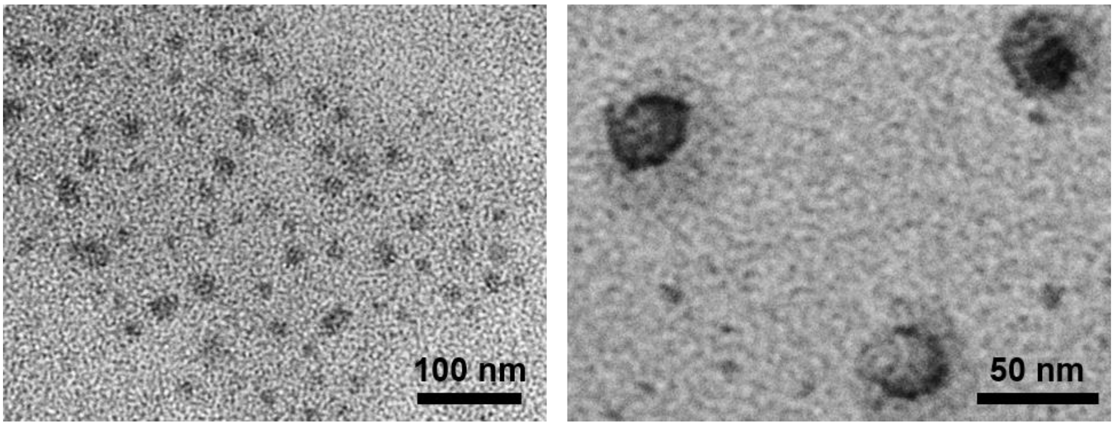

Figure S27. TEM images of the TBT-2(TP-DPA) nanoaggregates.

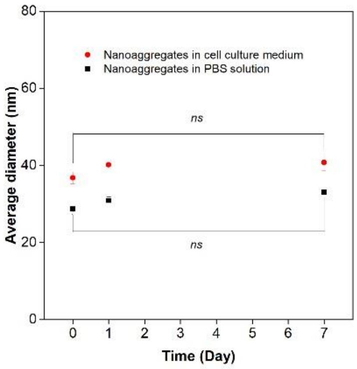

Figure S28. Physiological stability of the PTA nanoaggregates in 1× PBS and complete cell culture medium. The average diameters of the nanoaggregates in the cell culture medium and PBS were measured by dynamic laser scattering (DLS) after continuous incubation for 0, 1 and 7 days, separately.

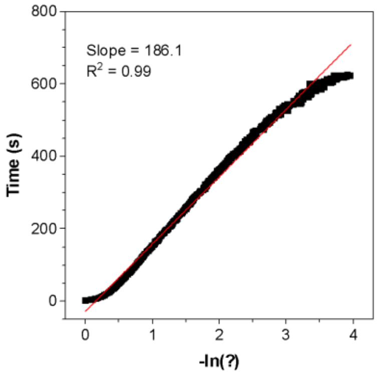

Figure S29. The calculation of photothermal conversion efficiency of TBT-2(TP-DPA) nanoaggregates. The PTA nanoaggregates (1 mg mL^{-1}) was upon 808 nm irradiation (1 W cm^{-2}) for 5 minutes, and then the laser was shut off. Time constant for heat transfer of PTA nanoaggregates was calculated to be τ_S = 186.1 s.

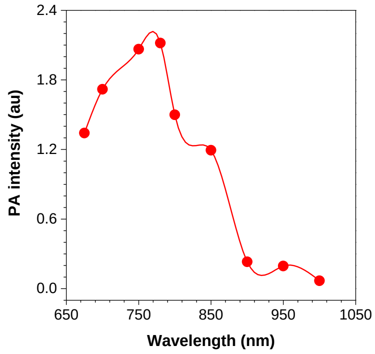

Figure S30. The photoacoustic spectrum of TBT-2(TP-DPA) nanoaggregates in aqueous solution.

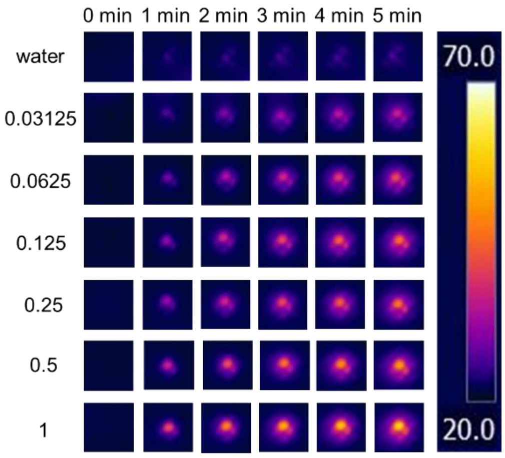

Figure S31. Photothermal performance of PTA nanoaggregates *in vitro*. IR thermal images of TBT-2(TP-DPA) nanoaggregates with different concentrations (0.03125-1 mg mL^{-1}) under an 808 nm laser irradiation (1 W cm^{-2}) for 5 min.

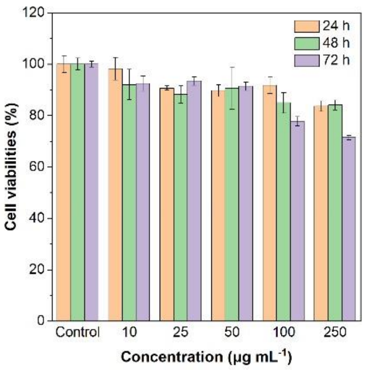

Figure S32. The viabilities of 4T1 cells after treatment with TBT-2(TP-DPA) nanoaggregates at concentrations of 0, 10, 25, 50, 100 and 250 μg mL^{-1} for 24 h. 48 h, and 72h.

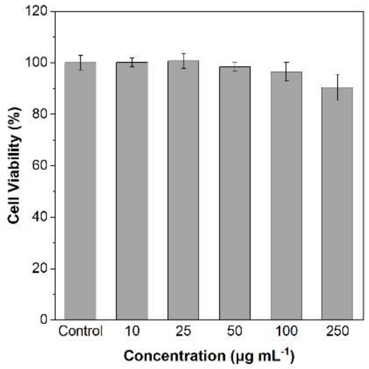

Figure S33. The viabilities of 293T cells after treatment with TBT-2(TP-DPA) nanoaggregates at concentrations of 0, 10, 25, 50, 100 and 250 μg mL^{-1} for 24 h.

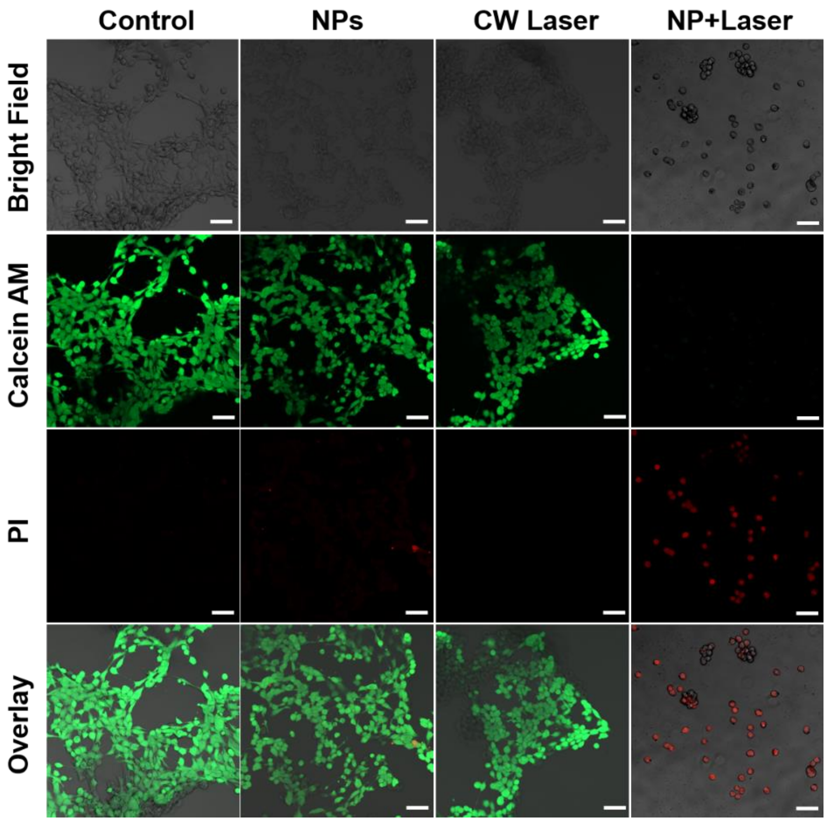

Figure S34. Live/dead assay results of 4T1 cells after varied treatments, including cells without treatment (control), PTA nanoaggregates-treated cells (NPs), 808 nm CW laser-treated cells (CW laser), and cells treated with PTA nanoaggregates and 808 nm CW laser (NP + Laser). Laser power: 1 W cm^{-2}. Scale bars: 50 μm.

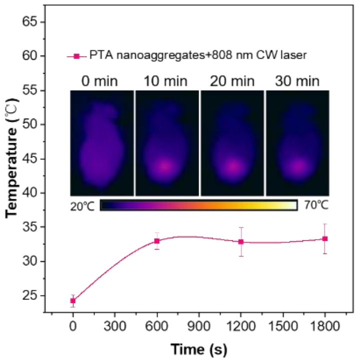

Figure S35. Photothermal performance of the mice tumor region when injecting PTA nanoaggregates and immediately irradiated by 808 nm CW laser. Insets: IR thermal images of PTA nanoaggregates with different irradiation time (1 W cm^{-2}).

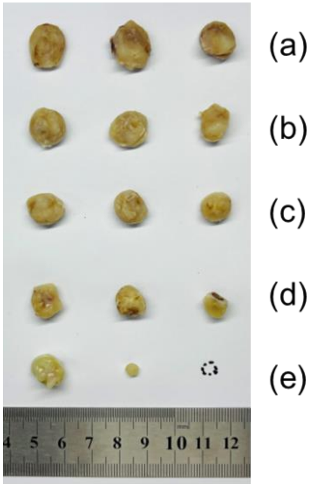

Figure S36. The digital images of the tumor on the 14th day with various treatments in photothermal therapy. Note: (a) control, (b) 808 nm pulse laser + 808 nm CW laser, (c) PTA, (d) EPR 24 h + PTT and (e) PTA 40 min+ PTT.

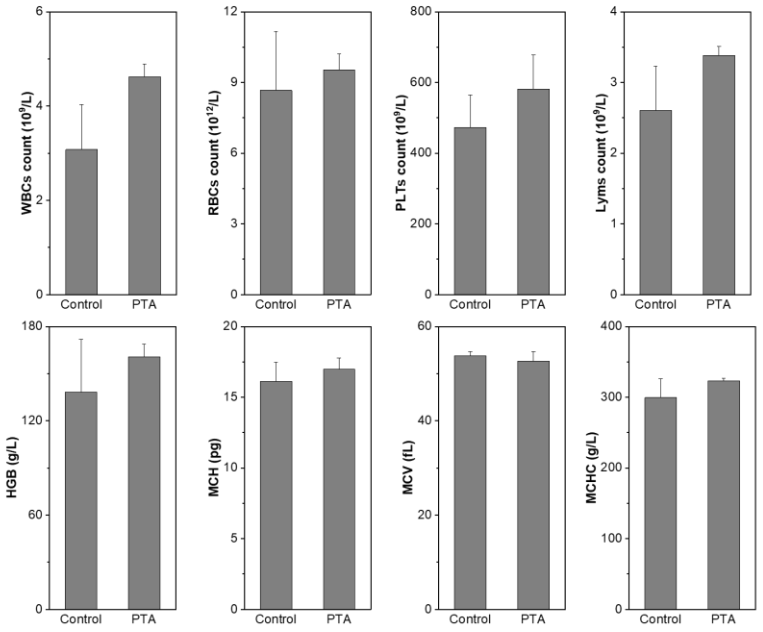

Figure S37. The *in vivo* biosafety analysis of PTA nanoaggregates. Blood routine analysis data, containing the numbers of WBC, RBC, PLT, Lym and HGB, MCH (mean corpsular hemoglobin), MCV (mean corpsular volume), MCHC (mean corpsular hemoglobin concentration) of control and PTA nanoaggregates treated mice.

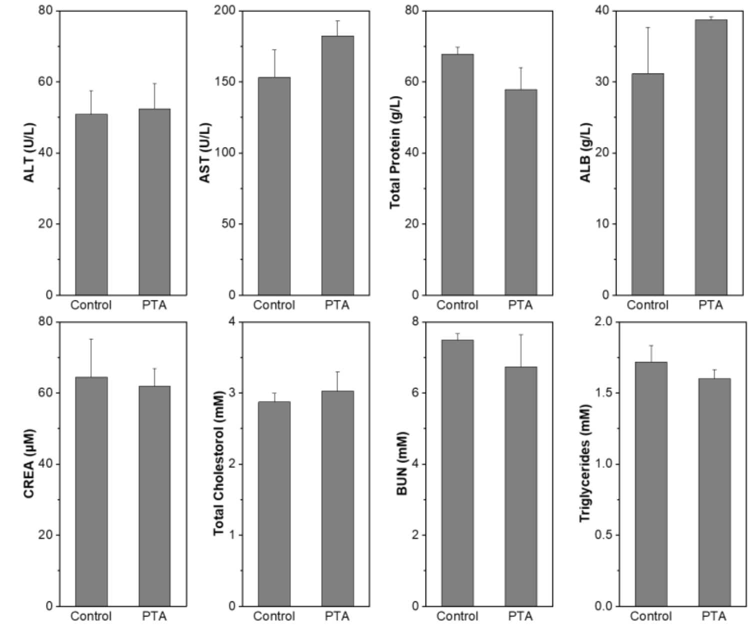

Figure S38. The *in vivo* biosafety analysis of PTA nanoaggregates. Serum biochemistry data, containing ALT, AST, total protein, ALB, creatinine (CREA), total

cholesterol, blood urea nitrogen (BUN) and triglycerides levels of control and PTA nanoaggregates treated mice were measured.

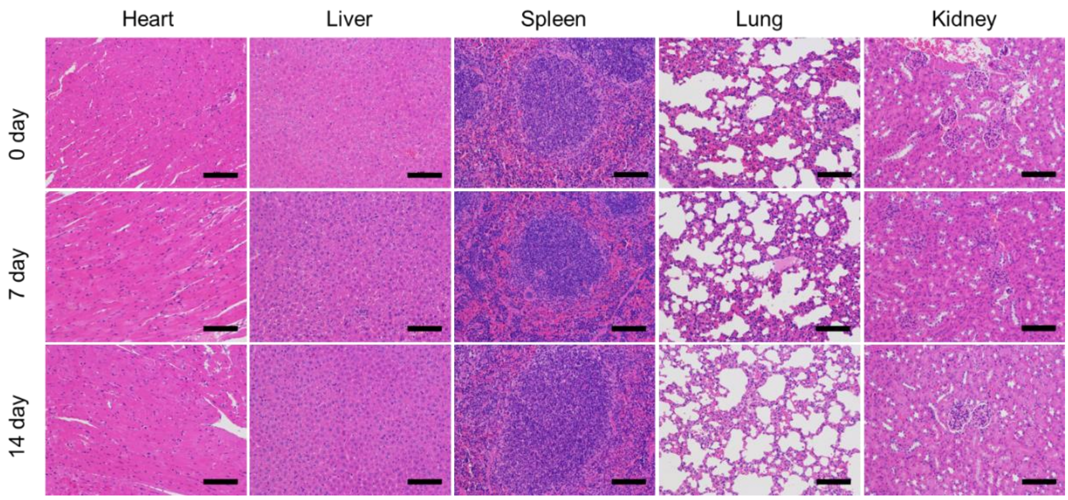

Figure S39. H&E-staining images of major organs (heart, liver, spleen, lung and kidney) of the mice collected from the control group and the PTA nanoaggregates-treated mice at 7 day and 14 day after intravenous injection of PTA nanoaggregates. Scale bars: 100 μm.

Table S1. Summary of Absolute QY of TBT-2(TP-DPA) in different solvents and solid state.

Solvent or solid state	QY (%)
Toluene	10.4
DCM	5.1
Chloroform	9.5
THF	6.8
DMF	~0.8
Solid	~1.9
